# Novel use of tranexamic acid to reduce the need for Nasal Packing in Epistaxis (NoPac) randomised controlled trial: research protocol

**DOI:** 10.1136/bmjopen-2018-026882

**Published:** 2019-02-15

**Authors:** Adam Reuben, Andrew Appelboam, Andy Barton, Patricia Jane Vickery, Richard Body, Malcolm Hilton, Jason Coppell, Paul Ewings

**Affiliations:** 1 Academic Department of Emergency Medicine, Royal Devon and Exeter Hospital NHS Foundation Trust, Exeter, UK; 2 Research Design Service South West, Bristol, UK; 3 Peninsula Clinical Trials Unit, Plymouth University, Plymouth, UK; 4 Department of Emergency Medicine, Manchester Royal Infirmary, Manchester, UK; 5 Department of Otolaryngology, Royal Devon and Exeter Hospital, NHS Foundation Trust, Exeter, UK; 6 Department of Haematology, Royal Devon and Exeter Hospital, NHS Foundation Trust, Exeter, UK

**Keywords:** epistaxis, tranexamic acid, topical, intranasal, packing, emergency

## Abstract

**Introduction:**

Patients presenting to emergency departments (EDs) with epistaxis uncontrolled by subsequent simple first aid measures or application of topical vasoconstrictors will typically undergo anterior nasal packing. Packing is effective, but can be extremely painful and unpleasant and patients usually need hospital admission. Tranexamic acid (TXA) is a cheap, safe, readily available antifibrinolytic agent known to be beneficial in a variety of clinical settings where uncontrolled bleeding may be a problem. Anecdotal evidence suggests that topical TXA may be of value in persistent epistaxis; however, further evaluation is required.

**Methods and analysis:**

This is a multicentre, double-blind, parallel group, randomised, controlled trial comparing the use of topical intranasal TXA with indistinguishable placebo in adults presenting to UK EDs with persistent atraumatic epistaxis. Follow-up is at 1 week by structured telephone review. The primary outcome measure is the subsequent need for anterior nasal packing in the ED. Key secondary outcomes include the need for hospital admission, blood transfusion and/or further treatment for epistaxis during the index ED attendance. Recruiting 450 patients will provide 90% power to demonstrate an absolute reduction in packing rate from 95% to 85%. An improvement of this magnitude would be of significant benefit to patients and healthcare providers and justify a change to standard practice. Given the low cost of TXA and its short administration time, a full economic evaluation is not being undertaken.

**Ethics and dissemination:**

The study has been approved by the South West—Bristol Research Ethics Committee (reference 17/SW/0010). We aim to publish the findings in a high impact, international peer-reviewed journal. Results will also be shared with the Hereditary Haemorrhagic Telangiectasia foundation and telangiectasia UK for dissemination through appropriate related forums.

**Trial registration number:**

ISRCTN34153772 and EudraCT No: 2016-001530-10.

**Strengths and limitations of this study**

- Atraumatic epistaxis is a common presentation to the emergency department.
- This is a randomised, double-blind, placebo-controlled study.
- The pragmatic study design ensures ease of recruitment and minimal deviation from standard practice.
- Recruitment to emergency department research studies is notoriously difficult due to workload and staffing pressures.
- The wide variety of approaches to treatment in different centres and by different practitioners may lead to difficulties in standardisation of management.

## Introduction

Epistaxis (nosebleed) is an extremely common condition which causes 60% of the population to seek medical attention at some point in their lives.^1^ Patients are frequently elderly, with the mean age of over 70 years.^2^ In most cases, epistaxis resolves with simple measures. These may include firm pressure with the thumb and index finger to the soft anterior part of the nose, the use of ice packs on the bridge of the nose and adopting a forward leaning posture. Many cases, however, are more serious, leading to hospital admission or even, occasionally, death.^3^ Current approaches to managing this condition in the emergency department (ED) beyond simple first aid include the use of topical vasoconstrictors, which have been shown to arrest bleeding in up to 65% of patients.^4^ Chemical cautery with silver nitrate may also be used but with profuse bleeds, it may be difficult to identify the bleeding site and successfully apply cautery.^5^ These initial measures may be partially successful and are occasionally repeated to stop the bleeding. If bleeding cannot be stopped with these measures, patients will usually undergo anterior nasal packing.

While effective, nasal packing is recognised to be extremely uncomfortable for patients. The mean pain score associated with insertion of the most commonly used nasal packs (the Merocel) has been shown to be higher than the average pain scores for patients with an acute myocardial infarction (heart attack).^6 7^ Nasal packs typically remain in situ for at least 24 hours, which causes ongoing pain (reported mean pain scores 0.5–3.5/10) and an uncomfortable sensation of nasal obstruction. One typical published patient story reports, ‘The packing had my face aching all night.’^8^ Removal is also painful with reported pain scores ranging from 0.4 to 7.4. In addition to the pain caused by this procedure, our unpublished feasibility data demonstrate that 86% of patients who undergo anterior nasal packing are admitted to hospital and patients have a mean length of stay of 2 days. Nasal packing is also associated with a complication rate of up to 28%.^9^ Reported complications include infection, toxic shock syndrome, hypoxia, sleep apnoea, pack displacement with airway obstruction and bleeding on removal.

A recent unpublished national survey (Florey E, Reuben A, Appelboam A, 2014) has shown significant variation in the ED treatments used, which vary according to local protocols, clinician experience, available expertise and departmental workload. Feasibility data at three centres suggest that one-third of patients presenting to the ED with epistaxis subsequently require nasal packing.

Tranexamic acid (TXA) is an antifibrinolytic agent which acts by competitively binding to both plasminogen and plasmin in circulating blood at the site of injury, reducing the production and action of plasmin. Plasmin promotes the breakdown of fibrin, a protein that forms the stabilising framework of blood clots, therefore by inhibiting the action of plasmin, clots become more stable. TXA may be used via the intravenous or oral routes, having systemic action, or topically, where its effects are very localised, acting on the mucosa/blood vessels with which it is in contact.

TXA has been used in a variety of clinical and research settings with good evidence of its general efficacy and safety.^10^ A large randomised controlled trial (RCT) including 20 000 patients showed that intravenous TXA reduces mortality in patients with major trauma and suspected bleeding.^11^ Importantly, in this large trial, TXA did not cause an increase in thromboembolic complications. In a meta-analysis of 129 trials including 10 488 patients, intravenous TXA was found to reduce mortality and the need for blood transfusion in patients undergoing surgery.^12^ No adverse events were noted in any of the patients receiving topical or intravenous treatment. When applied topically in patients undergoing surgery, TXA has been shown to reduce bleeding and the need for blood transfusion.^10^ The bioavailability of topical TXA is unknown (about 50% via gastrointestinal tract), but the systemic absorption of a topical dose of 200 mg (compared with the standard intravenous dose of 1 g) is extremely unlikely to result in significant side effects. Plasma concentrations following topical application are less than 1/10 of the level after intravenous administration.^10^ Results of an unpublished local study (Florey E, Appelboam A, Reuben A, 2015) to estimate the quantity of TXA absorbed through the nasal mucosa, suggested that of 200 mg applied to a dry dental roll, a maximum of 39.8 mg is absorbed systemically, with an average of just 14 mg. There are no absolute contraindications to the use of TXA and the large multicentre RCTs CRASH (Clinical Randmoisation of an Antifibrinolytic in Significant Haemorrhage)^11^ and HALT-IT (Haemorrhage Alleviation with tranexamic Acid - Initestinal System)^13^ have set a precedent with no notable exclusion criteria.

Topical application of TXA has the potential to inhibit local fibrinolysis at the site of bleeding with minimal systemic absorption.^10^ Our systematic review identified that two RCTs have evaluated topical TXA as a treatment for epistaxis.^14^ The first, conducted in 1995, randomised 68 patients to receive topical TXA gel or placebo gel and demonstrated non-significant trends towards lower rates of continued bleeding after 30 min and rebleeding within 30 days.^15^ However, the trial was underpowered to detect clinically important differences and significantly more patients who received TXA had presented with moderate or severe bleeds. The second trial randomised 216 patients to receive topical TXA (dental rolls soaked with 500 mg in 5 mL of TXA) or anterior nasal packing. Significantly, fewer patients in the TXA group had active bleeding after 10 min with an absolute risk reduction of 40% (number needed to treat 2.5) suggesting substantial benefit. However, this trial was limited by a potential lack of allocation concealment and was unblinded.^16^ While they have limitations, these trials suggest that topical TXA is a promising treatment for epistaxis, which could reduce the need for patients to undergo a painful, unpleasant procedure (anterior nasal packing) with its attendant complications and hospital admissions. Topical TXA for persistent epistaxis may, therefore, be both beneficial for the patient (reduced nasal packing and reduced hospital admission) and for the healthcare system (saving on bed occupancy and providing a cheaper alternative to packing and admission).

## Aims and objectives

The aim of the study is to test the effectiveness of topical intranasal TXA in reducing the need for anterior nasal packing in adult patients presenting to the ED with spontaneous atraumatic epistaxis.

Key objectives are to compare the effect of topical TXA versus placebo on the need for anterior nasal packing, any further treatment in the ED, hospital admission and subsequent length of hospital stay, the requirement for blood products, the rebleeding rate for patients subsequently discharged from the ED and any adverse events, including thrombotic complications.

## Methods and analysis

### Study design

**Figure 1:**
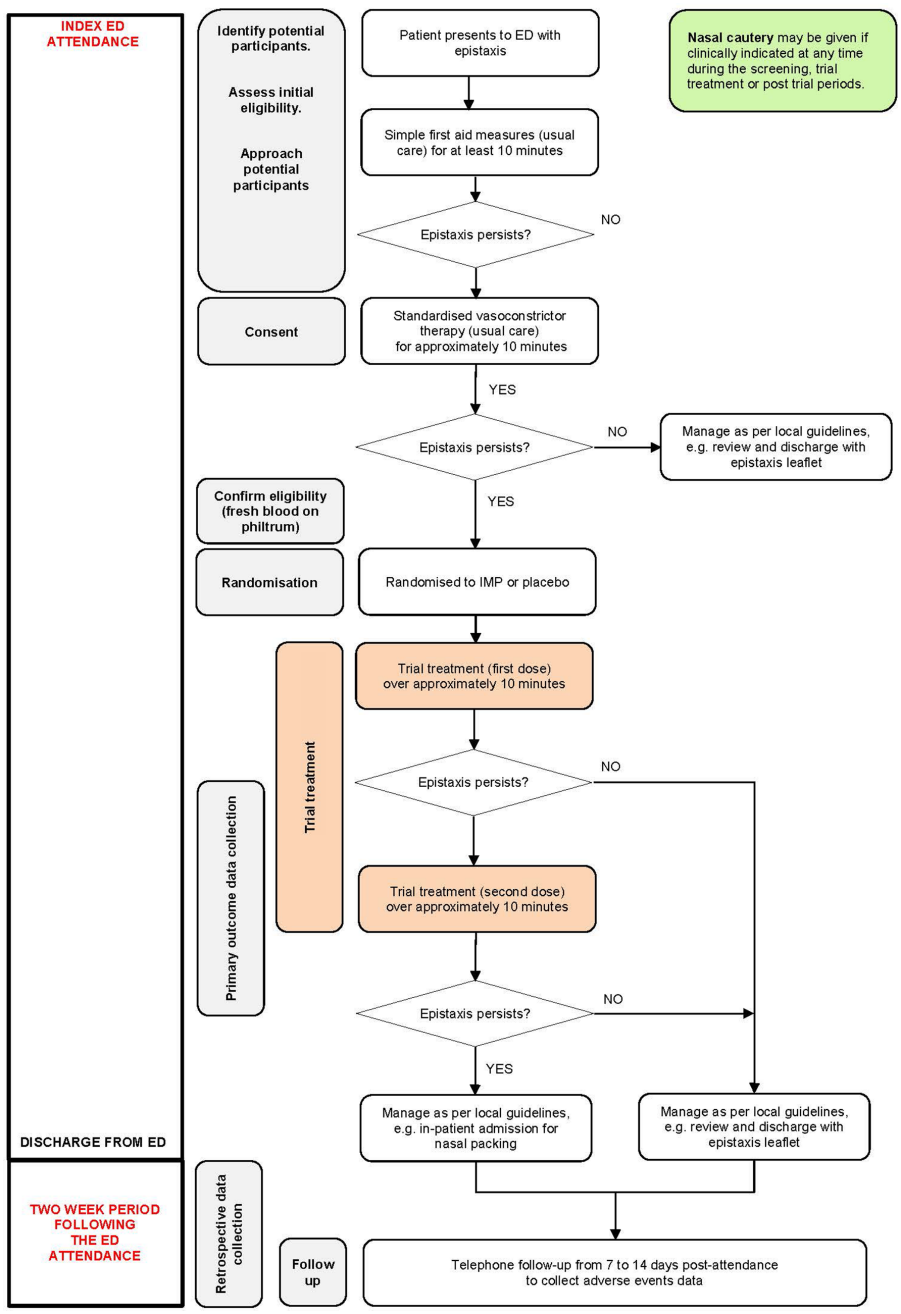
Participant pathway. ED, emergency department; IMP, investigational medicinal product.

This study is a pragmatic 1:1 individually randomised, double-blind, parallel group placebo controlled multicentre trial. Four hundred and fifty patients presenting to the ED with epistaxis and bleeding that persists after simple first aid measures, followed by standardised topical vasoconstrictor therapy will be randomised to receive either TXA or matched placebo (water for injection), soaked onto a cotton wool dental roll. Outcomes will include anterior nasal packing (primary), any further treatment for epistaxis during the index ED attendance, hospital admission, length of stay, need for blood products and rebleeding rates for patients discharged home. Thrombotic complications will also be monitored as this is a recognised side effect of TXA, although this is considered a negligible risk with the low-dose topical TXA used in this study. Figure 1 illustrates the participant pathway through the trial.

A standard operating procedure (SOP) for the initial management of epistaxis has been implemented at participating sites prior to the study opening to recruitment (online supplementary appendix 1). The purpose of this was to standardise (1) the application of digital pressure to compress the nose and (2) the use of a topical vasoconstrictor soaked on a cotton wool roll and gently inserted into the bleeding nostril. While not universally used, vasoconstrictor use has been shown to have utility in epistaxis and is advocated in national guidance.^6 17^ Participating sites are required to manage potential trial patients according to this standardised procedure for epistaxis management prior to confirming whether patients are eligible for inclusion in the trial. The current protocol is V.1.1, last updated on 2 September 2018.

10.1136/bmjopen-2018-026882.supp1 Supplementary data

### Study population and setting

The screening and recruitment of patients, delivery of the intervention and recording of outcomes will be carried out within participating UK National Health Service (NHS) EDs (online supplementary appendix 2).

10.1136/bmjopen-2018-026882.supp2 Supplementary data

### Screening, recruitment and consent

Adult patients (aged 18 or over) with acute spontaneous epistaxis and continued, uncontrolled bleeding after at least 10 min of simple first aid measures (eg, digital pressure) will be screened for eligibility. Unstable patients or patients with an indication to proceed to immediate nasal packing will be excluded. Full inclusion and exclusion criteria for the trial are summarised in box 1.

Box 1 **Inclusion and exclusion criteria**

**Inclusion criteria**

Potential participants must satisfy the following criteria to be enrolled in the study:

- Aged 18 or over.
- Presenting to the emergency department (ED) with spontaneous, atraumatic epistaxis, unresolved with simple first aid and standard initial therapy.

**Exclusion criteria**

Potential participants meeting any of the following criteria will be excluded from study participation:

- Clinical evidence of shock, as determined by the treating clinician or requirement for resuscitation (including but not limited to systolic blood pressure <90 mm Hg).
- Known allergy to tranexamic acid.
- Lacking capacity to give consent.
- Unwilling to give consent.
- No telephone or unwilling to be contacted by telephone.
- Known nasopharyngeal, nasal cavity or paranasal malignancy.
- Pregnancy.
- Sent to ED for specialist ear, nose, throat treatment.
- Already undergone prehospital nasal packing.
- Prior participation in the study (ie, received allocated treatment).
- Prisoners.
- Epistaxis caused by trauma (excluding simple nose picking).
- Known haemophilia.

Patients will be screened for eligibility by Good Clinical Practice (GCP) trained staff. ED attendance logs will be screened for the duration of the trial to enable the identification of any missed patients and reporting of simple demographic data to ensure there is no evidence of recruitment bias.

Potentially eligible patients will give written informed consent (online supplementary file) during 10 min of treatment with a topical vasoconstrictor (eg, phenylephrine or dilute epinephrine) soaked on a dental roll and standard nasal compression provided by a proprietary nasal clip, as directed by the SOP provided to participating sites. The written informed consent process will normally be undertaken by the attending clinician but may be delegated to another appropriate GCP-trained member of the research team depending on individual circumstances. Study team members nominated by the principal investigator to undertake the consent process will be listed as such on the study delegation log.

10.1136/bmjopen-2018-026882.supp3 Supplementary data

Potentially eligible patients will be provided with a concise participant information sheet (PIS), a verbal explanation of the purpose and nature of the trial and a description of what participation in the trial will entail. Patients will be given the opportunity to ask questions about the study. Patients providing consent and who continue to bleed after application of topical vasoconstrictor are confirmed eligible and will proceed to randomisation. Informed consent will be sought prior to confirmation of eligibility in order to prevent delay to further treatment should the patient continue to bleed from their nose once the vasoconstrictor-soaked dental roll is removed.

Most bleeding can be at least temporarily controlled with nasal compression sufficient to allow written informed consent. However, witness consent will be permitted for patients who are unable to provide written consent, for example, due to continued active bleeding during nasal compression but who have full capacity and have expressed a clear interest in taking part. If a patient agrees to participate, he/she will be asked to complete an informed consent form which will be countersigned by the staff member taking consent. A record of the patient’s consent to participate will be documented in the hospital notes where a copy of the completed consent form and PIS will also be filed.

### Randomisation and blinding

Treatment allocations will be determined by randomisation stratified by centre using variable sized blocks, from a computer-generated allocation sequence provided by the clinical trials unit (CTU). Sequentially numbered tamper-evident trial packs will be supplied by the manufacturer containing a sealed phial of the trial solution, two dental rolls and study-specific labels for the trial documentation and patient’s notes. Patients will be allocated to receive TXA or placebo by selection of the next sequentially numbered pack. Packs will be signed out by trained staff on the randomisation log. Appropriate sequential use of packs will be strictly audited by the site research teams, trial manager and trial management group.

Consideration was given to the possible need for stratification of patients according to anticoagulant use prior to randomisation. Local audit data from the Royal Devon and Exeter (RD&E) hospital have shown that packing rates for those on warfarin or one of the direct oral anticoagulants, as compared with those who are not, are within 5% of each other. Given the extra complexity that stratification would introduce in a time-pressured environment, and the likelihood that randomisation will achieve a good balance, no such stratification has been included.

This is a double-blind study hence neither the research teams responsible for treating and assessing patients within this trial nor the participants themselves are aware of individual treatment allocations. Should the clinical need arise, a procedure is in place to enable emergency unblinding of treatment allocation.

### Trial interventions

Treatments in both arms of the trial are provided by Stockport Pharmaceuticals who are licensed to manufacture products for clinical trials under a Medicines and Healthcare products Regulatory Agency (MHRA) Manufacturers Authorisation (investigational medicinal product, IMP) licence. Participants will receive either:

Tranexamic acid: Presented in a phial containing 4 mL of a 100 mg/mL solution, enough to saturate two standard cotton wool dental rolls. The standard preparation of TXA solution available in the UK contains 500 mg of drug in 5 mL of the clear, colourless solution. A cotton wool roll, such as is routinely used in the management of patients with epistaxis, will absorb approximately 2 mL (equivalent to 200 mg) of TXA.

Placebo: An identically presented phial containing 4 mL of the excipient used for the active IMP, that is, water for injection.

Packaging for trial treatments will be identical. A flag label on the phial will be affixed to the trial-specific data collection form for drug accountability purposes. Trial drugs will be stored, at ambient temperature, in a secure drug cupboard within the ED accessible only to trained staff. Approximately 2 mL of solution will be used to saturate a cotton wool dental roll which, once saturated, will be inserted into the patient’s nose. Sufficient solution will remain in the bottle to allow a second treatment according to the protocol.

The active and placebo solutions will be indistinguishable in appearance, taste and smell to patients and clinicians. All dental rolls will be left in situ for approximately 10 min, timed by the research nurse or attending clinician and accompanied by continued standardised nasal compression using a purpose designed disposable nasal clip. This is deemed to be a sufficient period for the formation and stabilisation of blood clots and hence control of bleeding. Patients, who deteriorate clinically during this period with uncontrolled bleeding or a fall in blood pressure, or where urgent nasal packing is clinically indicated, will have their dental roll removed and receive further treatment as directed clinically. In our experience, this would be a very rare event if the patient was initially stable and eligible for the trial. Such patients will be included in the intention-to-treat analyses.

Participants with ongoing bleeding after 10 min treatment with the IMP will undergo an identical second 10 min treatment using the remaining allocated solution as long as they remain eligible and willing to continue in the study.

Participants, who are eligible and randomised but not treated with at least one dose of IMP, for example, because the bleeding stopped between randomisation and administration of IMP, will also be included in the intention-to-treat analyses. However, participants randomised in error and in violation of the protocol, for example, when bleeding was not ongoing after vasoconstrictor therapy will be excluded.

### Primary outcome measure

The primary outcome will be the use of anterior nasal packing (of any type) at any time during that ED attendance. The need for packing will be determined after removal of the second trial dental roll by the treating clinician. Ongoing bleeding will be defined as the presence of visible blood below the nostril. Minor ongoing bleeding does not mandate nasal packing and the epistaxis management SOP will advise consideration of further measures such as silver nitrate cautery as appropriate. The decision to pack cannot be strictly defined and must be at the discretion of the clinician based on clinical assessment, but the blinding of clinicians will be sufficiently robust to prevent bias in this decision.

### Secondary outcome measures

Secondary outcome measures include hospital admission, need for blood transfusion, recurrent epistaxis and any thrombotic events requiring any hospital reattendance within 1 week. Any treatment during the index ED episode for continued bleeding and further ED hospital treatments required for epistaxis during the 7-day follow-up period will also be recorded, including details of the type of hospital episode.

### Data collection

Data required for the purposes of the trial will be recorded on a study-specific case report form (CRF) by the attending clinician, research nurse or other nominated research team member. To save time during the ED attendance, data will be retrospectively recorded in the CRF where applicable. At 1-week postattendance, the participant will be contacted by a member of the local research team to collect follow-up information including details of treatment for any further epistaxis and details of any other adverse events experienced since discharge.

### Postintervention management

Following a maximum of two doses of trial treatment, patients will be managed according to local departmental protocols. Any additional treatment(s) will be recorded in the CRF. Prior to discharge, all participants will be given a more detailed information sheet about the study.

### Safety monitoring

Extensive evidence supporting the use of both intravenous TXA (CRASH 2)^11^ and topical TXA^10^ (Cochrane review) in over 30 000 patients supports an excellent safety profile, with no reported complications in patients receiving topical TXA. The bioavailability of topical TXA is unknown (about 50% via GI tract), but the systemic absorption of a topical dose of 200 mg (compared with standard intravenous dose of 1 g) is extremely unlikely to result in significant side effects. The trial design excludes patients with haemodynamic instability, where the administration of trial treatments could delay urgent life-saving interventions.

Structured telephone review will allow monitoring of all potential complications. Particular attention will be paid to the possibility of thrombotic complications as this is a recognised side effect of both oral and intravenous TXA, although this risk is considered negligible with the low-dose topical TXA and the route used in this study.

All serious adverse events (SAEs) will be reported immediately, within 24 hours of discovery, to the CTU who will notify the chief investigator. All SAEs will be followed up until resolution. Any SAE considered to be related to trial treatment and not consistent with the information set out in the reference safety information for TXA will be classified as a suspected unexpected serious adverse reaction and details passed to the sponsor for immediate unblinding and expedited reporting to the MHRA, as required. In addition to SAEs, non-SAEs considered to be possibly, probably or definitely related to trial treatment will be recorded.

A summary report of all SAEs and non-SAEs will be scrutinised by members of the independent data monitoring committee (DMC), trial steering committee (TSC) and trial management group on a regular basis.

If an individual suffers negligent harm as a result of participating in the trial, NHS indemnity covers NHS staff and those people responsible for conducting the trial who have honorary contracts with the relevant NHS Trust. In the case of non-negligent harm, an ex gratia payment may be considered in the event of a claim. There are no specific provisions for ancillary or post-trial care.

### Data handling

Data will be collected and managed in accordance with the Data Protection Act 1998/General Data Protection Regulation 2018. Each participant will be allocated a unique study number and will then be identified in all study-related documentation by their identification number and initials.

Original CRFs will be posted to the CTU, with copies of the CRF retained at the study site. CRFs will be checked on receipt at CTU and any obvious errors or omissions rectified as far as possible by means of a formalised data query/clarification procedure. All data will be double entered by CTU staff on to a password-protected SQL Server database and encrypted using Secure Sockets Layer. Double-entered data will be compared for discrepancies using a stored procedure and discrepant data will be verified using the original CRF. After all data cleaning duties have been performed and the database locked, anonymised data will be exported to the trial statistician for blinded analysis.

### Determination of sample size

The null hypothesis for the trial is that there is no difference in anterior nasal packing rates for patients with persistent epistaxis after first aid and use of a topical vasoconstrictor, treated with either topical TXA or matched placebo. The patients recruited to the study will have failed simple first aid measures and vasoconstrictor and would ordinarily proceed to packing, according to local protocol. It is likely therefore that the need for packing in the control arm of the trial will be very high; a small number may have their bleeding stopped by the additional 10 min of dental roll insertion (although soaked in placebo), but we expect about 95% to need packing. We also expect that TXA might produce a large reduction in this need, but given it is inexpensive and safe, even a modest reduction would be worthwhile. A reduction from 95% to 85%, assuming a corrected χ^2^ test powered at 90% and with a significance level of 5%, requires 207 patients per group. Because of the nature and timing of the primary outcome, we do not envisage much loss to follow-up or missing data. To be conservative, we aim to recruit a total of 450 patients.

### Statistical analysis

A statistical analysis plan will be prepared by the trial statistician and approved by the DMC in advance of any analysis. The trial statistician will conduct the statistical analysis by nominal group (ie, blinded to treatment allocation) and present the results for interpretation by blinded members of the trial team and oversight committee, prior to disclosure of the treatment allocation and further interpretation of the study findings. There are no preplanned interim analyses.

Consolidated Standards of Reporting Trials guidelines^18^ will be used to present descriptive data on patients screened, recruited, randomised and treated, and any lost to follow-up. Descriptive data will be presented on baseline characteristics, for example, demographics. All primary comparative analyses will be based on the intention-to-treat principle, with consideration given to additional sensitivity analyses as appropriate considering per protocol populations (if different) and imputation for missing data. The outcomes (including, in particular, the primary outcome) are binary in nature and will be analysed by mixed effects logistic regression with centre as a random effect. All comparative analyses will be reported with point estimates (ORs), 95% CIs and p values.

### Missing data

Given the nature of the short follow-up and a limited amount of data to be collected, it is not anticipated that there will be many missing data. Every attempt will be made to retrieve any missing data. Where there data remain missing, sensitivity analyses may be conducted using a range of assumptions where necessary and consideration given to suitable imputation.

### Study management and oversight

The study sponsor organisation is the RD&E NHS Foundation Trust, Barrack Road, Exeter EX2 5DW. Day-to-day trial management is administered through the UKCRC (clinical research collaboration)-registered Peninsula CTU at Plymouth University. A trial management group including the chief investigator, CTU trial managers, trial statistician and other personnel relevant to the study (eg, clinicians, clinical trials pharmacist, CTU data manager, patient and sponsor representatives) will meet regularly (usually monthly) throughout the duration of the trial to oversee practical management of the trial.

A TSC, chaired by an independent member, will oversee the conduct and safety of the trial, ensuring that milestones are achieved and general scientific probity is maintained. An independent DMC will monitor the safety and ethics of the trial by overseeing recruitment, primary outcome data completeness and accumulating safety data.

### Patient and public involvement

A patient advisory group was established to contribute to the design of the study from the outset, including input into the proposed trial interventions. This group was made of individuals with prior experience of epistaxis requiring hospital treatment. Further to this, public involvement is maintained through patient representation on the trial management group and TSC. Results will be disseminated to study participants via a website hosted by the CTU and to a wider patient group via Hereditary Haemorrhagic Telangiectasia (CureHHT).

### Ethics and dissemination

The trial complies with the Declaration of Helsinki and GCP guidelines. All eligible, willing participants will undergo full written informed consent by GCP-trained staff before taking any part in the study. All protocol modifications will be reviewed by the study sponsor and communicated to relevant parties once approved.

The results will be applicable and of interest to emergency physicians, paramedics, acute physicians, NHS Trusts, general practitioners, emergency care practitioners and patients and efforts will be made to disseminate the information to all of these groups. To that end, the study will be submitted for publication in international, high impact, peer-reviewed journals whose target audience includes appropriate clinicians. Results will also be shared with the HHT foundation and telangiectasia UK (patient groups at significant risk of epistaxis) for publication on their websites and presentation through appropriate related forums. After the end of the study, information collected during the study may be made available as an anonymised participant-level dataset to other researchers under an appropriate data sharing agreement.

## Discussion

The Nasal Packing in Epistaxis study is a multicentre RCT designed to investigate the value of topical intranasal TXA for patients presenting to an ED with epistaxis that persists after simple first aid measures. Such patients frequently progress to requiring anterior nasal packing. While effective, packing is not a benign procedure, can be extremely painful, invariably requires hospital admission and has side effects and potential significant complications. TXA is safe, cheap and readily available.

A pragmatic design is intended to enhance, rather than impede, the patient journey. The study protocol follows an SOP for the management of epistaxis, which all recruiting centres have adopted in advance of the trial opening. Given the unpredictable, busy and challenging nature of the ED environment, all appropriate steps have been taken to ensure that the trial processes are simple and straightforward to complete. The trial has been specifically designed to meet its objectives and recruitment targets in this challenging ED setting, using a carefully considered effect size and accrual rate based on data from participating sites. The trial interventions will not interfere with the standard ED management of epistaxis, so patients will not in anyway be disadvantaged by their inclusion.

If TXA was found to be a useful adjunct to the management of persistent epistaxis, it could obviate the need for nasal packing in those patients where it is effective and reduce the need for hospital admission. This would be an important finding for the benefit of patients and healthcare systems.

Anterior nasal packing is an effective, but an uncomfortable method for controlling persistent epistaxis that usually requires hospital admission. Topical TXA has been shown to be safe and effective in a number of settings and there is some evidence that it may be of value in selected patients with epistaxis, although the question is yet to be fully evaluated. The results of this study could realistically lead to a reduction in the need for anterior nasal packing and thus hospital admission for patients with epistaxis and the reduction in for an uncomfortable procedure with recognised complications.

## Supplementary Material

Reviewer comments

Author's manuscript
