# Supplementary material for: Novel use of tranexamic acid to reduce the need for Nasal Packing in Epistaxis (NoPac) randomised controlled trial: research protocol
Source: BMJ Open. 2019 Feb 15;9(2):e026882. doi: 10.1136/bmjopen-2018-026882 (PMC6398761; doi:10.1136/bmjopen-2018-026882)
Supplement: Supplementary data [file bmjopen-2018-026882supp001.pdf]

Appendix 1: Epistaxis Standard Operating Procedure (SOP)

This haemostasis SOP is for use in cooperative, haemodynamically stable patients with *ongoing* bleeding. If in doubt, get senior help. See full ED guideline for details.

STEP 1
Ensure appropriate simple first aid measures have been tried. These have usually been undertaken pre-hospital or after triage.

↓

STEP 2
Clear any large blood clot from the nasal passages by gentle nose blowing, suction or with nasal forceps and examine the nose.

↓

STEP 3
If active bleeding, insert a dental roll soaked in **Lignocaine and Phenylephrine*** topical solution in the anterior nose and ask the patient to compress the nose over Little's area (soft part of nose) for 10 minutes with firm digital pressure.

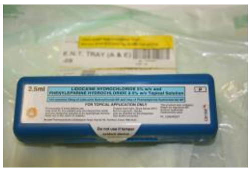

↓ 10 mins

STEP 4
Re-examine nose ideally with a nasal speculum and good light. Cauterize any visible culprit vessel in Little's area with silver nitrate.
Note: Generally significant bleeding needs to be controlled before cautery.
Minor continued bleeding may require further period of compression with the Lignocaine and Phenylephrine topical solution soaked roll.

↓

STEP 5
If on-going brisk bleeding despite above steps anterior pack the nose with either a rapid rhino or nasal tampon and refer to ENT

*or lignocaine and adrenaline or an alternative topical vasoconstrictor (according to preferred local protocols)
