# Supplementary material for: Novel use of tranexamic acid to reduce the need for Nasal Packing in Epistaxis (NoPac) randomised controlled trial: research protocol
Source: BMJ Open. 2019 Feb 15;9(2):e026882. doi: 10.1136/bmjopen-2018-026882 (PMC6398761; doi:10.1136/bmjopen-2018-026882)
Supplement: Supplementary data [file bmjopen-2018-026882supp002.pdf]

Appendix 2: NoPac study sites

Site Name
Royal Devon & Exeter Hospital, Exeter
Derriford Hospital, Plymouth
Royal United Hospital, Bath
Manchester Royal Infirmary
Gloucestershire Royal Hospital
Cheltenham General Hospital
Southmead Hospital, Bristol
North Devon District Hospital, Barnstaple
Musgrove Park Hospital, Taunton
Salford Royal Hospital
Royal Derby Hospital
Royal Infirmary of Edinburgh
Dorset County Hospital, Dorchester
Royal Cornwall Hospital, Truro
Norfolk & Norwich University Hospital, Norwich
Yeovil District Hospital
St George’s Hospital, London
St Thomas’ Hospital, London
John Radcliffe Hospital, Oxford
Barts Health NHS Trust: Royal London Hospital
Barts Health NHS Trust: Whipps Cross Hospital
Epsom & St Helier NHS Trust: Epsom Hospital
Epsom & St Helier NHS Trust: St Helier Hospital
Addenbrooke’s Hospital, Cambridge
Royal Berkshire Hospital, Reading
University Hospitals Coventry & Warwickshire
