# Supplementary material for: Novel use of tranexamic acid to reduce the need for Nasal Packing in Epistaxis (NoPac) randomised controlled trial: research protocol
Source: BMJ Open. 2019 Feb 15;9(2):e026882. doi: 10.1136/bmjopen-2018-026882 (PMC6398761; doi:10.1136/bmjopen-2018-026882)
Supplement: Supplementary data [file bmjopen-2018-026882supp003.pdf]

NHS Trust Header

Participant study number: | | | | | |

Principal Investigator: Dr <PI name>

PARTICIPANT CONSENT FORM

NoPac study: Novel use of tranexamic acid to reduce the need for nasal packing

Please initial each box

1. I confirm that I have read the information sheet (version 2.0 dated 20.02.2017) for the above study. I have had the opportunity to consider the information, ask questions and have had these answered satisfactorily. ☐

2. I understand that my participation in this study is voluntary and that I am free to withdraw at any time without giving any reason and without my medical care or legal rights being affected. ☐

3. I understand that relevant sections of my medical notes and information collected during the study may be looked at by individuals from the Sponsor organisation or its' representatives, my local NHS Trust and the regulatory authorities, where it is relevant to my taking part in this research. I give permission for these individuals to have access to my records. ☐

4. I agree to my General Practitioner being informed of my participation in the study. ☐

5. I agree that my telephone contact details may be temporarily stored at my local NHS Trust to allow the study team to contact me after one week. ☐

6. I consent to take part in this study. ☐

____________________ ________________ ____________________

Print name (Participant) Date Signature

IF PARTICIPANT IS UNABLE TO COMPLETE THIS FORM, ASK WITNESS TO COMPLETE THE FOLLOWING:

____________________ ________________ ____________________

Print name (Witness) Date Signature

Relationship of witness to participant: ____

____________________ ________________ ____________________

Print name (Person taking consent) Date Signature

When completed: 1 (original) for researcher site file; 1 copy for participant; 1 copy for medical notes
