# Supplementary material for: Novel use of tranexamic acid to reduce the need for Nasal Packing in Epistaxis (NoPac) randomised controlled trial: research protocol
Source: BMJ Open. 2019 Feb 15;9(2):e026882. doi: 10.1136/bmjopen-2018-026882 (PMC6398761; doi:10.1136/bmjopen-2018-026882)
Supplement: Author's manuscript [file bmjopen-2018-026882.draft_revisions.pdf]

BMJ Open is committed to open peer review. As part of this commitment we make the peer review history of every article we publish publicly available.

When an article is published we post the peer reviewers' comments and the authors' responses online. We also post the versions of the paper that were used during peer review. These are the versions that the peer review comments apply to.

The versions of the paper that follow are the versions that were submitted during the peer review process. They are not the versions of record or the final published versions. They should not be cited or distributed as the published version of this manuscript.

BMJ Open is an open access journal and the full, final, typeset and author-corrected version of record of the manuscript is available on our site with no access controls, subscription charges or pay-per-view fees (http://bmjopen.bmj.com).

If you have any questions on BMJ Open's open peer review process please email editorial.bmjopen@bmj.com

BMJ Open

The Novel Use of Tranexamic Acid to Reduce the Need for Nasal Packing in Epistaxis (NoPac) Randomised Controlled Trial: research protocol

Journal:	*BMJ Open*
Manuscript ID	bmjopen-2018-026882
Article Type:	Protocol
Date Submitted by the Author:	24-Sep-2018

Complete List of Authors:	Reuben, Adam; Royal Devon & Exeter Hospitals NHS Foundation Trust, Emergency Medicine<br>Appelboam, Andrew; Royal Devon & Exeter Hospital, Emergency Department<br>Barton, Andy; South West Research Design Service,<br>Vickery, Patricia; Plymouth University, Peninsula Clinical Trials Unit<br>Body, Richard; Manchester Royal Infirmary, Emergency Medicine<br>Hilton, Malcolm; Royal Devon and Exeter NHS Foundation Trust, Otolaryngology<br>Coppell, Jason; Royal Devon & Exeter Hospitals NHS Foundation Trust, Haematology<br>Ewings, Paul; Research Design Service, Research Office

Keywords:	Epistaxis, Tranexamic acid, Topical, Intranasal, Packing, Emergency

SCHOLARONE™
Manuscripts

The Novel Use of Tranexamic Acid to Reduce the Need for Nasal Packing in Epistaxis (NoPac) Randomised Controlled Trial: research protocol

Adam Reuben[1], Andrew Appelboam[1], Andy Barton[2], Jane Vickery[3], Richard Body[4], Malcolm Hilton[5], Jason Coppell[6], Paul Ewings[2]

[1]Academic Department of Emergency Medicine, Royal Devon and Exeter Hospital NHS Foundation Trust, Exeter; [2]Research Design Service South West; [3]Peninsula Clinical Trials Unit, Plymouth University;[4]Department of Emergency Medicine, Manchester Royal Infirmary, Manchester; [5]Department of Otolaryngology, Royal Devon and Exeter Hospital, NHS Foundation Trust; [6]Department of Haematology, Royal Devon and Exeter Hospital, NHS Foundation Trust.

Corresponding Author: Adam Reuben.

c/o Emergency Department, Royal Devon & Exeter Hospital NHS Foundation Trust, Barrack Road, Exeter, Devon EX2 5DW

Tel : 00 44 (0)1392 402085

Fax : 00 44 (0)1392 402313

Email: adam.reuben@nhs.net

For peer review only

For peer review only - http://bmjopen.bmj.com/site/about/guidelines.xhtml

Abstract

Introduction: Patients presenting to Emergency Departments (EDs) with epistaxis uncontrolled by subsequent simple first aid measures or application of topical vasoconstrictors will typically undergo anterior nasal packing. Packing is effective, but can be extremely painful and unpleasant and patients usually need hospital admission. Tranexamic Acid (TXA) is a cheap, safe, readily available antifibrinolytic agent known to be beneficial in a variety of clinical settings where uncontrolled bleeding may be a problem. Anecdotal evidence suggests that topical TXA may be of value in persistent epistaxis; however, further evaluation is required.

Methods and analysis: This is a multicentre, double-blind, parallel group, randomised, controlled trial comparing the use of topical intranasal TXA with indistinguishable placebo in adults presenting to UK EDs with persistent atraumatic epistaxis. Follow-up is at one week by structured telephone review. The primary outcome measure is the subsequent need for anterior nasal packing in the ED. Key secondary outcomes include need for hospital admission, blood transfusion and/or further treatment for epistaxis during the index ED attendance. Recruiting 450 patients will provide 90% power to demonstrate an absolute reduction in packing rate from 95% to 85%. An improvement of this magnitude would be of significant benefit to patients and health care providers and justify a change to standard practice. Given the low cost of TXA and its short administration time, a full economic evaluation is not being undertaken.

Ethics and dissemination: The study has been approved by the South West - Bristol Research Ethics Committee (reference 17/SW/0010). We aim to publish the findings in a high impact, international peer reviewed journal. Results will also be shared with the Hereditary Haemorrhagic Telangiectasia foundation (CureHHT) and telangiectasia UK for dissemination through appropriate related forums.

Registration: The study is registered with Current Controlled Trials (ISRCTN34153772) and the EU Clinical Trials Register (EudraCT No: 2016-001530-10).

Key words

Epistaxis; tranexamic acid; topical; intranasal; packing; emergency

For peer review only

For peer review only - http://bmjopen.bmj.com/site/about/guidelines.xhtml

Strengths and limitations of this study

- Common condition under scrutiny
- Pragmatic study design to ensure ease of recruitment and minimal deviation away from standard practice
- Double-blinded
- Emergency Department recruitment to research notoriously difficult due to workload and staffing pressures
- Wide variety in approaches to treatment in different centres and by different practitioners may lead to difficulties in standardisation

Introduction

Epistaxis (nosebleed) is an extremely common condition which causes 60% of the population to seek medical attention at some point in their lives[1]. Patients are frequently elderly, with mean age of over 70 years[2]. In most cases epistaxis resolves with simple measures. These may include firm pressure with the thumb and index finger to the soft anterior part of the nose, the use of ice packs on the bridge of the nose and adopting a forward leaning posture. Many cases, however, are more serious, leading to hospital admission or even, occasionally, death[3]. Current approaches to managing this condition in the Emergency Department (ED) beyond simple first aid, include the use of topical vasoconstrictors, which have been shown to arrest bleeding in up to 65% of patients[4]. Chemical cautery with silver nitrate may also be used but with profuse bleeds it may be difficult to identify the bleeding site and successfully apply cautery[5]. These initial measures may be partially successful and are occasionally repeated to stop the bleeding. If bleeding cannot be stopped with these measures, patients will usually undergo anterior nasal packing.

Whilst effective, nasal packing is recognised to be extremely uncomfortable for patients. The mean pain score associated with insertion of the most commonly used nasal packs (the Merocel) has been shown to be higher than the average pain scores for patients with an acute myocardial infarction (heart attack)[6,7]. Nasal packs typically remain in situ for at least 24 hours, which causes on-going pain (reported mean pain scores 0.5 – 3.5/10) and an uncomfortable sensation of nasal obstruction. One typical published patient story reports, “The packing had my face aching all night”[8]. Removal is also painful with reported pain scores ranging from 0.4 to 7.4. In addition to the pain caused by this procedure, our unpublished feasibility data demonstrate that 86% of patients who undergo anterior nasal packing are admitted to hospital and patients have a mean length of stay of two days. Nasal packing is also associated with a complication rate of up to 28%[9]. Reported complications include infection, toxic shock syndrome, hypoxia, sleep apnoea, pack displacement with airway obstruction, and bleeding on removal.

A recent unpublished national survey (Florey E, Reuben A, Appelboam A, 2014) has shown significant variation in the ED treatments used, which vary according to local protocols, clinician experience, available expertise and departmental workload. Feasibility data at three centres suggest that one third of patients presenting to the ED with epistaxis subsequently require nasal packing.

For peer review only - http://bmjopen.bmj.com/site/about/guidelines.xhtml

TXA is an antifibrinolytic agent which acts by competitively binding to both plasminogen and plasmin in circulating blood at the site of injury, reducing the production and action of plasmin. Plasmin promotes the breakdown of fibrin, a protein that forms the stabilising framework of blood clots, therefore by inhibiting the action of plasmin, clots become more stable. TXA may be used via the intravenous or oral routes, having systemic action, or topically, where its effects are very localised, acting on the mucosa / blood vessels with which it is in contact.

TXA has been used in a variety of clinical and research settings with good evidence of its general efficacy and safety[10]. A large RCT including 20,000 patients showed that intravenous TXA reduces mortality in patients with major trauma and suspected bleeding[11]. Importantly, in this large trial TXA did not cause an increase in thromboembolic complications. In a meta-analysis of 129 trials including 10,488 patients, intravenous TXA was found to reduce mortality and the need for blood transfusion in patients undergoing surgery[12]. No adverse events were noted in any of the patients receiving topical or intravenous treatment. When applied *topically* in patients undergoing surgery, TXA has been shown to reduce bleeding and the need for blood transfusion[10]. The bioavailability of topical TXA is unknown (about 50% via GI tract), but the systemic absorption of a topical dose of 200mg (compared with standard IV dose of 1g) is extremely unlikely to result in significant side effects. Plasma concentrations following topical application are less than one tenth of the level after intravenous administration[10]. Results of an unpublished local study (Florey E, Appelboam A, Reuben A, 2015) to estimate the quantity of TXA absorbed through the nasal mucosa, suggested that of 200mg applied to a dry dental roll, a maximum of 39.8mg is absorbed systemically, with an average of just 14mg. There are no absolute contra-indications to the use of TXA and the large multi-centre RCTs CRASH[11] and HALT-IT[13] have set a precedent with no notable exclusion criteria.

Topical application of tranexamic acid has the potential to inhibit local fibrinolysis at the site of bleeding with minimal systemic absorption[10]. Our systematic review identified that two randomised controlled trials have evaluated topical TXA as a treatment for epistaxis[14]. The first, conducted in 1995, randomised 68 patients to receive topical TXA gel or placebo gel and demonstrated non-significant trends towards lower rates of continued bleeding after 30 minutes and re-bleeding within 30 days[15]. However, the trial was underpowered to detect clinically important differences and significantly more patients who received TXA had presented with moderate or severe bleeds. The second trial randomised 216 patients to receive topical TXA (dental rolls soaked with 500mg in 5ml of TXA) or anterior nasal packing. Significantly fewer patients in the TXA group had active bleeding after 10 minutes with an absolute risk reduction of 40% (number needed to treat 2.5) suggesting substantial benefit. However, this trial was limited by a potential lack of allocation concealment and was unblinded[16]. While they have limitations, these trials suggest that topical TXA is a promising treatment for epistaxis, which could reduce the need for patients to undergo a painful, unpleasant procedure (anterior nasal packing) with its attendant complications and hospital admissions. Topical TXA for persistent epistaxis may therefore be both beneficial for the patient (reduced nasal packing and reduced hospital admission) and for the healthcare system (saving on bed occupancy and providing a cheaper alternative to packing and admission).

For peer review only - http://bmjopen.bmj.com/site/about/guidelines.xhtml

Aims and objectives

The aim of the study is to test the effectiveness of topical intranasal TXA in reducing the need for anterior nasal packing in adult patients presenting to the ED with spontaneous atraumatic epistaxis.

Key objectives are to compare the effect of topical TXA versus placebo on the need for anterior nasal packing, any further treatment in the ED, hospital admission and subsequent length of hospital stay, the requirement for blood products, the re-bleeding rate for patients subsequently discharged from the ED and any adverse events, including thrombotic complications.

Methods and analysis

Study design

This study is a pragmatic 1:1 individually randomised, double-blind, parallel group placebo controlled multi-centre trial. Four hundred and fifty patients presenting to the ED with epistaxis and bleeding that persists after simple first aid measures, followed by standardised topical vasoconstrictor therapy will be randomised to receive either TXA or matched placebo (water for injection), soaked onto a cotton wool dental roll. Outcomes will include anterior nasal packing (primary), any further treatment for epistaxis during the index ED attendance, hospital admission, length of stay, need for blood products and re-bleeding rates for patients discharged home. Thrombotic complications will also be monitored as this is a recognised side effect of TXA, although this is considered a negligible risk with the low dose topical TXA used in this study. Figure 1 illustrates the participant pathway through the trial.

A Standard Operating Procedure (SOP) for the initial management of epistaxis has been implemented at participating sites prior to the study opening to recruitment (Appendix 1). The purpose of this was to standardise i) the application of digital pressure to compress the nose and ii) the use of a topical vasoconstrictor soaked on a cotton wool roll and gently inserted into the bleeding nostril. Whilst not universally used, vasoconstrictor use has been shown to have utility in epistaxis and is advocated in national guidance[6,17]. Participating sites are required to manage potential trial patients according to this standardised procedure for epistaxis management prior to confirming whether patients are eligible for inclusion in the trial.

Study population and setting

The screening and recruitment of patients, delivery of the intervention and recording of outcomes will be carried out within participating UK NHS EDs (Appendix 2).

Screening, recruitment and consent

Adult patients (aged 18 or over) with acute spontaneous epistaxis and continued, uncontrolled bleeding after at least 10 minutes of simple first aid measures (e.g. digital pressure) will be screened for eligibility. Unstable patients or patients with an indication to proceed to immediate nasal packing, will be excluded. Full inclusion and exclusion criteria for the trial are summarised in Table 1.

For peer review only - http://bmjopen.bmj.com/site/about/guidelines.xhtml

Table 1: Inclusion and exclusion criteria

**Inclusion criteria**
Potential participants must satisfy the following criteria to be enrolled in the study:<br>• Aged 18 or over<br>• Presenting to the ED with spontaneous, atraumatic epistaxis, unresolved with simple first aid and standard initial therapy
**Exclusion criteria**
Potential participants meeting any of the following criteria will be excluded from study participation:<br>• Clinical evidence of shock, as determined by the treating clinician, or requirement for resuscitation (including but not limited to systolic BP<90 mmHg).<br>• Known allergy to TXA<br>• Lacking capacity to give consent<br>• Unwilling to give consent<br>• No telephone or unwilling to be contacted by telephone<br>• Known nasopharyngeal, nasal cavity or paranasal malignancy<br>• Pregnancy<br>• Sent to ED for specialist ENT treatment<br>• Already undergone pre-hospital nasal packing<br>• Prior participation in the study (i.e. received allocated treatment)<br>• Prisoners<br>• Epistaxis caused by trauma (excluding simple nose picking)<br>• Known haemophilia

Patients will be screened for eligibility by Good Clinical Practice (GCP) trained staff. ED attendance logs will be screened for the duration of the trial to enable the identification of any missed patients and reporting of simple demographic data to ensure there is no evidence of recruitment bias.

Potentially eligible patients will give written informed consent during 10 minutes of treatment with a topical vasoconstrictor (e.g. phenylephedrine or dilute adrenaline) soaked on a dental roll and standard nasal compression provided by a proprietary nasal clip, as directed by the SOP provided to participating sites. The written informed consent process will normally be undertaken by the attending clinician but may be delegated to another appropriate GCP-trained member of the research team depending on individual circumstances. Study team members nominated by the Principal Investigator to undertake the consent process will be listed as such on the study delegation log.

Potentially eligible patients will be provided with a concise Participant Information Sheet (PIS), a verbal explanation of the purpose and nature of the trial and a description of what

For peer review only - http://bmjopen.bmj.com/site/about/guidelines.xhtml

participation in the trial will entail. Patients will be given the opportunity to ask questions about the study. Patients providing consent and who continue to bleed after application of topical vasoconstrictor are confirmed eligible and will proceed to randomisation. Informed consent will be sought prior to confirmation of eligibility in order to prevent delay to further treatment should the patient continue to bleed from their nose once the vasoconstrictor-soaked dental roll is removed.

Most bleeding can be at least temporarily controlled with nasal compression sufficient to allow written informed consent. However, witness consent will be permitted for patients who are unable to provide written consent e.g. due to continued active bleeding during nasal compression but who have full capacity and have expressed clear interest in taking part. If a patient agrees to participate, he/she will be asked to complete an Informed Consent Form which will be countersigned by the staff member taking consent. A record of the patient's consent to participate will be documented in the hospital notes where a copy of the completed consent form and participant information sheet will also be filed.

Randomisation and blinding

Treatment allocations will be determined by randomisation stratified by centre using variable sized blocks, from a computer-generated allocation sequence provided by the CTU. Sequentially numbered tamper-evident trial packs will be supplied by the manufacturer containing a sealed vial of the trial solution, two dental rolls and study-specific labels for the trial documentation and patient's notes. Patients will be allocated to receive TXA or placebo by selection of the next sequentially numbered pack. Packs will be signed out by trained staff on the randomisation log. Appropriate sequential use of packs will be strictly audited by the site research teams, trial manager and Trial Management Group.

Consideration was given to the possible need for stratification of patients according to anticoagulant use prior to randomisation. Local audit data from the RD&E hospital have shown that packing rates for those on warfarin or one of the direct oral anti-coagulants (DOACs), as compared with those who are not, are within 5% of each other. Given the extra complexity that stratification would introduce in a time-pressured environment, and the likelihood that randomisation will achieve good balance, no such stratification has been included.

This is a double-blind study hence neither the research teams responsible for treating and assessing patients within this trial, nor the participants themselves, are aware of individual treatment allocations. Should the clinical need arise, a procedure is in place to enable emergency unblinding of treatment allocation.

Trial interventions

Treatments in both arms of the trial are provided by Stockport Pharmaceuticals who are licensed to manufacture products for clinical trials under a Medicines and Healthcare products Regulatory Agency (MHRA) Manufacturers Authorisation (IMP) licence. Participants will receive either:

For peer review only

For peer review only - http://bmjopen.bmj.com/site/about/guidelines.xhtml

a) tranexamic acid: presented in a vial containing 4mls of a 100mg/ml solution, enough to saturate two standard cotton wool dental rolls. The standard preparation of TXA solution available in the UK contains 500mg of drug in 5mls of clear, colourless solution. A cotton wool roll, such as is routinely used in the management of patients with epistaxis, will absorb approximately 2mls (equivalent to 200mg) of TXA.

b) placebo: an identically presented vial containing 4mls of the excipient used for the active investigational medicinal product (IMP) i.e. water for injection.

Packaging for trial treatments will be identical. A flag label on the vial will be affixed to the trial-specific data collection form for drug accountability purposes. Trial drugs will be stored, at ambient temperature, in a secure drug cupboard within the ED accessible only to trained staff. Approximately 2mls of solution will be used to saturate a cotton wool dental roll which, once saturated, will be inserted into the patient's nose. Sufficient solution will remain in the bottle to allow a second treatment according to the protocol.

The active and placebo solutions will be indistinguishable in appearance, taste and smell to patients and clinicians. All dental rolls will be left in situ for approximately 10 minutes, timed by the research nurse or attending clinician and accompanied by continued standardised nasal compression using a purpose designed disposable nasal clip. This is deemed to be a sufficient period for the formation and stabilisation of blood clots and hence control of bleeding. Patients who deteriorate clinically during this period with uncontrolled bleeding or a fall in blood pressure, or where urgent nasal packing is clinically indicated, will have their dental roll removed and receive further treatment as directed clinically. In our experience this would be a very rare event if the patient was initially stable and eligible for the trial. Such patients will be included in the intention-to-treat analyses.

Participants with on-going bleeding after 10 minutes' treatment with the IMP will undergo an identical second 10 minute treatment using the remaining allocated solution as long as they remain eligible and willing to continue in the study.

Participants who are eligible and randomised but not treated with at least one dose of IMP e.g. because the bleeding stopped between randomisation and administration of IMP, will also be included in the intention-to-treat analyses. However, participants randomised in error and in violation of the protocol e.g. when bleeding was not on-going after vasoconstrictor therapy, will be excluded.

Primary outcome measure

The primary outcome will be the use of anterior nasal packing (of any type) at any time during that ED attendance. The need for packing will be determined after removal of the second trial dental roll by the treating clinician. On-going bleeding will be defined as the presence of visible blood below the nostril. Minor on-going bleeding does not mandate nasal packing and the epistaxis management SOP will advise consideration of further measures such as silver nitrate cautery as appropriate. The decision to pack cannot be strictly defined and must be at the discretion of the clinician based on clinical assessment, but the blinding of clinicians will be sufficiently robust to prevent bias in this decision.

Secondary outcome measures

For peer review only - http://bmjopen.bmj.com/site/about/guidelines.xhtml

Secondary outcome measures include hospital admission, need for blood transfusion, recurrent epistaxis and any thrombotic events requiring any hospital re-attendance within one week. Any treatment during the index ED episode for continued bleeding and further ED hospital treatments required for epistaxis during the 7 day follow-up period will also be recorded, including details of the type of hospital episode.

Data collection

Data required for the purposes of the trial will be recorded on a study specific case report form (CRF) by the attending clinician, research nurse or other nominated research team member. To save time during the ED attendance, data will be retrospectively recorded in the CRF where applicable. At one week post-attendance, the participant will be contacted by a member of the local research team to collect follow-up information including details of treatment for any further epistaxis and details of any other adverse events experienced since discharge.

Post-intervention management

Following a maximum of two doses of trial treatment, patients will be managed according to local departmental protocols. Any additional treatment(s) will be recorded in the CRF. Prior to discharge, all participants will be given a more detailed information sheet about the study.

Safety monitoring

Extensive evidence supporting the use of both IV TXA (CRASH 2)[11] and topical TXA[10] (Cochrane review) in over 30,000 patients supports an excellent safety profile, with no reported complications in patients receiving topical TXA. The bioavailability of topical TXA is unknown (about 50% via GI tract), but the systemic absorption of a topical dose of 200mg (compared with standard IV dose of 1g) is extremely unlikely to result in significant side-effects. The trial design excludes patients with haemodynamic instability, where the administration of trial treatments could delay urgent life-saving interventions.

Structured telephone review will allow monitoring of all potential complications. Particular attention will be paid to the possibility of thrombotic complications as this is a recognised side effect of both oral and intravenous TXA, although this risk is considered negligible with the low dose topical TXA and the route used in this study.

All serious adverse events (SAEs) will be reported immediately, within 24 hours of discovery, to the CTU who will notify the Chief Investigator. All SAEs will be followed up until resolution. Any SAE considered to be related to trial treatment and not consistent with the information set out in the reference safety information for tranexamic acid will be classified as a suspected unexpected serious adverse reaction (SUSAR) and details passed to the sponsor for immediate unblinding and expedited reporting to the MHRA, as required. In addition to SAEs, non-serious adverse events considered to be possibly, probably or definitely related to trial treatment will be recorded.

A summary report of all serious and non-serious adverse events will be scrutinised by members of the independent Data Monitoring Committee (DMC), Trial Steering Committee and Trial Management Group on a regular basis.

For peer review only - http://bmjopen.bmj.com/site/about/guidelines.xhtml

Data handling

Data will be collected and managed in accordance with the Data Protection Act 1998 / General Data Protection Regulation 2018. Each participant will be allocated a unique study number and will then be identified in all study-related documentation by their identification number and initials.

Original CRFs will be posted to the CTU, with copies of the CRF retained at the study site. CRFs will be checked on receipt at CTU and any obvious errors or omissions rectified as far as possible by means of a formalised data query/clarification procedure. All data will be double-entered by CTU staff on to a password-protected SQL Server database and encrypted using Secure Sockets Layer. Double-entered data will be compared for discrepancies using a stored procedure and discrepant data will be verified using the original CRF. After all data cleaning duties have been performed and the database locked, anonymised data will be exported to the trial statistician for blinded analysis.

Determination of sample size

The null hypothesis for the trial is that there is no difference in anterior nasal packing rates for patients with persistent epistaxis after first aid and use of a topical vasoconstrictor, treated with either topical TXA or matched placebo. The patients recruited to the study will have failed simple first aid measures and vasoconstrictor and would ordinarily proceed to packing, according to local protocol. It is likely therefore that the need for packing in the control arm of the trial will be very high; a small number may have their bleeding stopped by the additional 10 minutes of dental roll insertion (albeit soaked in placebo), but we expect about 95% to need packing. We also expect that TXA might produce a large reduction in this need, but given it is inexpensive and safe, even a modest reduction would be worthwhile. A reduction from 95% to 85%, assuming a corrected chi-squared test powered at 90% and with a significance level of 5%, requires 207 patients per group. Because of the nature and timing of the primary outcome, we do not envisage much loss to follow-up or missing data. To be conservative, we aim to recruit a total of 450 patients.

Statistical analysis

A Statistical Analysis Plan will be prepared by the trial statistician and approved by the DMC in advance of any analysis. The trial statistician will conduct the statistical analysis by nominal group (i.e. blinded to treatment allocation) and present the results for interpretation by blinded members of the trial team and oversight committee, prior to disclosure of the treatment allocation and further interpretation of the study findings. There are no pre-planned interim analyses.

CONSORT guidelines[18] will be used to present descriptive data on patients screened, recruited, randomised and treated, and any loss to follow-up. Descriptive data will be presented on baseline characteristics, e.g. demographics. All primary comparative analyses will be based on the intention-to-treat principle, with consideration given to additional sensitivity analyses as appropriate considering per protocol populations (if different) and imputation for missing data. The outcomes (including in particular the primary outcome) are binary in nature and will be analysed by mixed effects logistic regression with centre as a random effect. All comparative analyses will be reported with point estimates (odds ratios), 95% confidence intervals and p-values.

For peer review only - http://bmjopen.bmj.com/site/about/guidelines.xhtml

Missing data

Given the nature of the short follow-up and limited amount of data to be collected, it is not anticipated that there will be many missing data. Every attempt will be made to retrieve any missing data. Where there data remain missing, sensitivity analyses may be conducted using a range of assumptions where necessary and consideration given to suitable imputation.

Study management and oversight

The study sponsor organisation is the Royal Devon and Exeter NHS Foundation Trust, Barrack Road, Exeter EX2 5DW. Day to day trial management is administered through the UKCRC-registered Peninsula Clinical Trials Unit at Plymouth University. A Trial Management Group including the Chief Investigator, CTU trial managers, trial statistician and other personnel relevant to the study (e.g. clinicians, clinical trials pharmacist, CTU data manager, patient and Sponsor representatives) will meet regularly (usually monthly) throughout the duration of the trial to oversee practical management of the trial.

A Trial Steering Committee (TSC), chaired by an independent member, will oversee the conduct and safety of the trial, ensuring that milestones are achieved and general scientific probity is maintained. An independent Data Monitoring Committee (DMC) will monitor the safety and ethics of the trial by overseeing recruitment, primary outcome data completeness and accumulating safety data.

Ethics and dissemination

The trial complies with Declaration of Helsinki and GCP guidelines. The study has been approved by the South West – Central Bristol Research Ethics Committee (REC reference 17/SW/0010) and each of the participating NHS Trust's Research and Development departments. All eligible, willing participants will undergo full written informed consent by GCP-trained staff before taking any part in the study.

The results will be applicable and of interest to emergency physicians, paramedics, acute physicians, NHS Trusts, general practitioners, emergency care practitioners and patients and efforts will be made to disseminate the information to all of these groups. To that end, the study will be submitted for publication in international, high impact, peer reviewed journals whose target audience includes appropriate clinicians. Results will also be shared with the Hereditary Haemorrhagic Telangiectasia (HHT) foundation and telangiectasia UK (patient groups at significant risk of epistaxis) for publication on their websites and presentation through appropriate related forums. After the end of the study, information collected during the study may be made available as an anonymised participant level dataset to other researchers under an appropriate data sharing agreement.

Discussion

The NoPac study is a multi-centre randomised controlled trial designed to investigate the value of topical intranasal tranexamic acid for patients presenting to an ED with epistaxis that persists after simple first aid measures. Such patients frequently progress to requiring anterior nasal packing. Whilst effective, packing is not a benign procedure, can be

For peer review only - http://bmjopen.bmj.com/site/about/guidelines.xhtml

extremely painful, invariably requires hospital admission and has side-effects and potential significant complications. TXA is safe, cheap and readily available.

A pragmatic design is intended to enhance, rather than impede, the patient journey. The study protocol follows a standard operating procedure for the management of epistaxis, which all recruiting centres have adopted in advance of the trial opening. Given the unpredictable, busy and challanging nature of the ED environment, all appropriate steps have been taken to ensure that the trial processes are simple and straight forward to complete. The trial has been specifically designed to meet its objectives and recruitment targets in this challenging ED setting, using a carefully considered effect size and accrual rate based upon data from participating sites. The trial interventions will not interfere with the standard ED management of epistaxis, so patients will not in anyway be disadvantaged by their inclusion.

If TXA were found to be a useful adjunct to the management of persistent epistaxis, it could obviate the need for nasal packing in those patients where it is effective and reduce the need for hospital admission. This would be an important finding for the benefit of patients and healthcare systems.

Anterior nasal packing is an effective, but uncomfortable method for controlling persistent epistaxis that usually requires hospital admission. Topical TXA has been shown to be safe and effective in a number of settings and there is some evidence that it may be of value in selected patients with epistaxis, although the question is yet to be fully evaluated. The results of this study could realistically lead to a reduction in the need for anterior nasal packing and thus hospital admission for patients with epistaxis and the reduction in for an uncomfortable procedure with recognised complications.

Acknowledgments

We would like to acknowledge and thank Wendy Ingram and Alison Jeffery, Peninsula Clinical Trials Unit, for all their hard work and administrative skills in support of this project and Ian Veitch, our patient representative, for his help and advice since the study start.

Funding

This research is funded by the NIHR Research for Patient Benefit Programme (project number PB-PG-0215-36142). The views expressed are those of the author(s) and not necessarily those of the NHS, the NIHR or the Department of Health. The funder has no role in study design or the collection, management, analysis or interpretation of data.

Patient and public involvement

A Patient Advisory Group was established to contribute to the design of the study from the outset, including input into the proposed trial interventions. This group was made of individuals with prior experience of epistaxis requiring hospital treatment. Further to this, public involvement is maintained through patient representation on the Trial Management Group and TSC. Results will be disseminated to study participants via a website hosted by the CTU and to a wider patient group via CureHHT.

For peer review only

For peer review only - http://bmjopen.bmj.com/site/about/guidelines.xhtml

Trial Management Group

Dr Adam Reuben (Chief Investigator); Mr Andy Barton (Associate Professor in Healthcare Research Methodology, Plymouth University); Prof Andrew Appelboam (Co-applicant, Honorary Associate Clinical Professor, University of Exeter Medical School), Professor Paul Ewings (Trial Statistician; Director, SW Research Design Service), Ms Jane Vickery (Senior Trial Manager, Peninsula Clinical Trials Unit), Dr Jason Coppell (Co-applicant, Consultant Haematologist, Royal Devon & Exeter NHS Foundation Trust), Mr Malcolm Hilton (Co-applicant, Consultant Otolaryngologist, Royal Devon & Exeter NHS Foundation Trust), Prof Richard Body (Co-applicant, Professor of Emergency Medicine, Central Manchester University Hospitals NHS Foundation Trust, Dr Alison Kerridge (Sponsor representative, Royal Devon & Exeter NHS Foundation Trust), Mrs Fiona Walters (Pharmacy Clinical Trials Manager, Royal Devon & Exeter NHS Foundation Trust), Mrs Shirley Todd (pharmacist), Mr Ian Veitch (Patient and Public Involvement representative), Gayle Githens-Mazer (Research Delivery Manager, NIHR Clinical Research Network South West Peninsula).

Trial Steering Committee

Chair, Prof Jonathan Benger (Consultant in Emergency Medicine, University Hospitals Bristol NHS Foundation Trust); Dr Adam Reuben (Chief Investigator, Consultant in Emergency Medicine, Royal Devon & Exeter Hospital); Mr Richard Williams (Specialist Registrar in Ear, Nose and Throat Surgery, Bristol Royal Infirmary) Dr Fiona Warren (Research Fellow in Statistics, University of Exeter Medical School), Prof Andrew Green (Patient and Public Involvement Representative)

Independent Data Monitoring Committee

Professor Gordon Taylor; Dr Tim Nokes (Consultant Haematologist, Derriford Hospital, Plymouth); Professor Tim Coates (Consultant Emergency Medicine, Leicester Royal Infirmary; Professor Paul Ewings (Research Design Service SW)

Competing Interests

There are no competing interests to declare.

Contributorship statement

All authors were co-applicants on the NIHR RfPB grant application, helped with the design of the study and have contributed to successive drafts of this paper.

AR is the Chief Investigator, provides clinical expertise and was responsible for conceiving the study and contributing to its initial design.

AA provides clinical expertise, contributed to the initial design of the study and supports the ongoing management of the study.

JV is the trial manager, responsible for overseeing the day-to-day running of the study.

PE is the trial statistician and provided expertise in the overall design of the study.

For peer review only

For peer review only - http://bmjopen.bmj.com/site/about/guidelines.xhtml

RB provides clinical expertise, contributed to the initial design of the study and supports the ongoing management of the study.

MH provides clinical expertise in otolaryngology and contributes to study management.

JC provides clinical expertise in haematology and contributes to study management.

AB provides methodological expertise and contributes to study management.

Representatives of the study sponsor attend trial management meetings and have reviewed the study protocol.

For peer review only

For peer review only - http://bmjopen.bmj.com/site/about/guidelines.xhtml

References

1. Pope LER, Hobbs CGL. Epistaxis: an update on current management. Postgrad Med J 2005;81:309-314

2. Traboulsi H, Alam E, Hadi U. Changing Trends in the Management of Epistaxis. *Int J Otolaryngol*. 2015. 2015:263987

3. Tan LK, Calhoun KH. Epistaxis. *Med Clin North Am*. 1999;83:43–56

4. Krempl, G. A. and A. D. Noorily (1995). Use of oxymetazoline in the management of epistaxis. Ann Otol Rhinol Laryngol 104(9 Pt 1): 704-6

5. Barr GD. Silver nitrate cautery and epistaxis. Arch Emerg Med 1989;6:233

6. Pringle MB, Beasley P, Brightwell AP. The use of Merocel nasal packs in the treatment of epistaxis. J Laryngol Otol 2006:96;543-546

7. Herlitz J, Richterova A, Bondestam E. Chest pain in acute myocardial infarction: a descriptive study according to subjective assessment and morphine requirement. Clin Cardiol 1986;9:423-428

8. http://www.emedicinehealth.com/nosebleeds/viewer-comments_em-74-page3.htm emedicine health viewer comments

9. Germann CA, Southall JC. Management of epistaxis and complications associated with anterior nasal packing. Ann Emerg Med 2004;44:s43-s44

10. Ker K, Beecher D, Roberts I. Topical application of tranexamic acid for the reduction of bleeding (review). Cochrane Database Syst Rev 2013;7: CD010562

11. The CRASH-2 Study. The CRASH-2 Collaborators. Lancet 2010;377:1096-1101

12. Ker K, Prieto-Merino D, Roberts I. Systematic review, meta-analysis and meta-regression of the effect of tranexamic acid on surgical blood loss. B J Surg 2013;100(10)

13. Roberts I, Coates T, Edwards P. HALT-IT - tranexamic acid for the treatment of gastrointestinal bleeding: study protocol for a randomised controlled trial Published online 2014 Nov 19. doi: 10.1186/1745-6215-15-450

14. Hilton L, Reuben AD. Topical intranasal tranexamic acid for spontaneous epistaxis. BESTBETS Best evidence topic reviews http://bestbets.org/bets/bet.php?id=2569

15. Tibbelin A, Aust R, Bende M et al. Effect of local tranexamic acid gel in the treatment of epistaxis ORL 1995;57:207-209

16. Zahed R, Moharamzadeh P, AlizadehArasi S. A new and rapid method for epistaxis treatment using injectable form of tranexamic acid topically: a randomized controlled trial 2013;31:1389-1392

17. https://bestpractice.bmj.com/topics/en-gb/421?q=Epistaxis&c=suggested

18. http://www.consort-statement.org/

For peer review only

For peer review only - http://bmjopen.bmj.com/site/about/guidelines.xhtml

Figure 1: Participant pathway

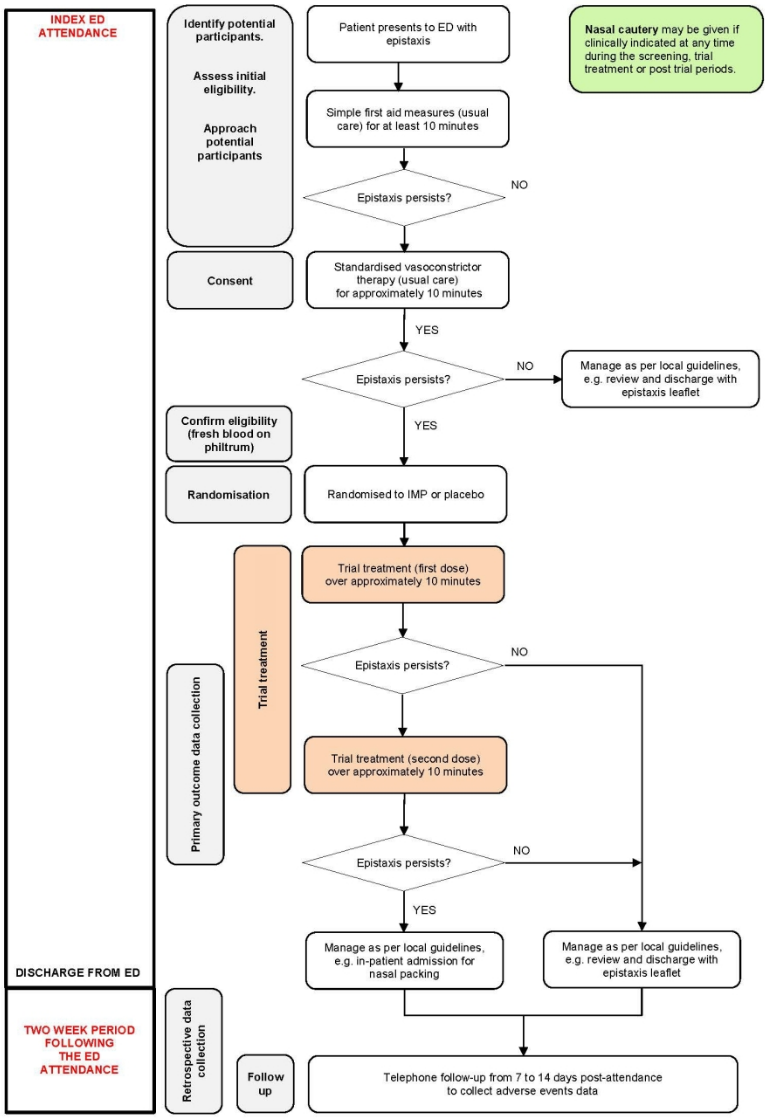

209x297mm (200 x 200 DPI)

Appendix 1: Epistaxis Standard Operating Procedure (SOP)

This haemostasis SOP is for use in cooperative, haemodynamically stable patient with *ongoing* bleeding. If in doubt, get senior help. See full ED guideline for details.

STEP 1
Ensure appropriate simple first aid measures have been tried. These have usually been undertaken pre-hospital or after triage.

↓

STEP 2
Clear any large blood clot from the nasal passages by gentle nose blowing, suction or with nasal forceps and examine the nose.

↓

STEP 3
If active bleeding, insert a dental roll soaked in **Lignocaine and Phenylephrine*** topical solution in the anterior nose and ask the patient to compress the nose over Little's area (soft part of nose) for 10 minutes with firm digital pressure.

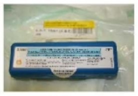

↓ 10 mins

STEP 4
Re-examine nose ideally with a nasal speculum and good light. Cauterize any visible culprit vessel in Little's area with silver nitrate.
Note: Generally significant bleeding needs to be controlled before cautery.
Minor continued bleeding may require further period of compression with the Lignocaine and Phenylephrine topical solution soaked roll.

↓

STEP 5
If on-going brisk bleeding despite above steps anterior pack the nose with either a rapid rhino or nasal tampon and refer to ENT

210x297mm (200 x 200 DPI)

Appendix 2: NoPac study sites

Site Name
Royal Devon & Exeter Hospital, Exeter
Derriford Hospital, Plymouth
Royal United Hospital, Bath
Manchester Royal Infirmary
Gloucestershire Royal Hospital
Cheltenham General Hospital
Southmead Hospital, Bristol
North Devon District Hospital, Barnstaple
Musgrove Park Hospital, Taunton
Salford Royal Hospital
Royal Derby Hospital
Royal Infirmary of Edinburgh
Dorset County Hospital, Dorchester
Royal Cornwall Hospital, Truro
Norfolk & Norwich University Hospital, Norwich
Yeovil District Hospital
St George's Hospital, London
St Thomas' Hospital, London
John Radcliffe Hospital, Oxford
Barts Health NHS Trust: Royal London Hospital
Barts Health NHS Trust: Whipps Cross Hospital
Epsom & St Helier NHS Trust: Epsom Hospital
Epsom & St Helier NHS Trust: St Helier Hospital
Addenbrooke's Hospital, Cambridge
Royal Berkshire Hospital, Reading
University Hospitals Coventry & Warwickshire

For peer review only

STANDARD PROTOCOL ITEMS: RECOMMENDATIONS FOR INTERVENTIONAL TRIALS

SPIRIT 2013 Checklist: Recommended items to address in a clinical trial protocol and related documents*

Section/item	Item No	Description	Addressed on page number
**Administrative information**			
Title	1	Descriptive title identifying the study design, population, interventions, and, if applicable, trial acronym	______1______
Trial registration	2a	Trial identifier and registry name. If not yet registered, name of intended registry	______2______
	2b	All items from the World Health Organization Trial Registration Data Set	______________
Protocol version	3	Date and version identifier	Not included
Funding	4	Sources and types of financial, material, and other support	______12_____
Roles and responsibilities	5a	Names, affiliations, and roles of protocol contributors	____1, 13-14__
	5b	Name and contact information for the trial sponsor	_____11______
	5c	Role of study sponsor and funders, if any, in study design; collection, management, analysis, and interpretation of data; writing of the report; and the decision to submit the report for publication, including whether they will have ultimate authority over any of these activities	__12, 13, 14___
	5d	Composition, roles, and responsibilities of the coordinating centre, steering committee, endpoint adjudication committee, data management team, and other individuals or groups overseeing the trial, if applicable (see Item 21a for data monitoring committee)	_____11, 13___

For peer review only - http://bmjopen.bmj.com/site/about/guidelines.xhtml

Introduction

Background and rationale	6a	Description of research question and justification for undertaking the trial, including summary of relevant studies (published and unpublished) examining benefits and harms for each intervention	_____3, 4_____
	6b	Explanation for choice of comparators	_____5_______
Objectives	7	Specific objectives or hypotheses	______5______
Trial design	8	Description of trial design including type of trial (eg, parallel group, crossover, factorial, single group), allocation ratio, and framework (eg, superiority, equivalence, noninferiority, exploratory)	______5______

Methods: Participants, interventions, and outcomes

Study setting	9	Description of study settings (eg, community clinic, academic hospital) and list of countries where data will be collected. Reference to where list of study sites can be obtained	_____5_______
Eligibility criteria	10	Inclusion and exclusion criteria for participants. If applicable, eligibility criteria for study centres and individuals who will perform the interventions (eg, surgeons, psychotherapists)	______6______
Interventions	11a	Interventions for each group with sufficient detail to allow replication, including how and when they will be administered	______7,8______
	11b	Criteria for discontinuing or modifying allocated interventions for a given trial participant (eg, drug dose change in response to harms, participant request, or improving/worsening disease)	______8_______
	11c	Strategies to improve adherence to intervention protocols, and any procedures for monitoring adherence (eg, drug tablet return, laboratory tests)	_____N/A______
	11d	Relevant concomitant care and interventions that are permitted or prohibited during the trial	_____N/A______
Outcomes	12	Primary, secondary, and other outcomes, including the specific measurement variable (eg, systolic blood pressure), analysis metric (eg, change from baseline, final value, time to event), method of aggregation (eg, median, proportion), and time point for each outcome. Explanation of the clinical relevance of chosen efficacy and harm outcomes is strongly recommended	______8,9______
Participant timeline	13	Time schedule of enrolment, interventions (including any run-ins and washouts), assessments, and visits for participants. A schematic diagram is highly recommended (see Figure)	______6 -9____

For peer review only - http://bmjopen.bmj.com/site/about/guidelines.xhtml

Sample size	14	Estimated number of participants needed to achieve study objectives and how it was determined, including clinical and statistical assumptions supporting any sample size calculations	_____10______
Recruitment	15	Strategies for achieving adequate participant enrolment to reach target sample size	Not included
**Methods: Assignment of interventions (for controlled trials)**			
Allocation:			
Sequence generation	16a	Method of generating the allocation sequence (eg, computer-generated random numbers), and list of any factors for stratification. To reduce predictability of a random sequence, details of any planned restriction (eg, blocking) should be provided in a separate document that is unavailable to those who enrol participants or assign interventions	_____7_______
Allocation concealment mechanism	16b	Mechanism of implementing the allocation sequence (eg, central telephone; sequentially numbered, opaque, sealed envelopes), describing any steps to conceal the sequence until interventions are assigned	_____7_______
Implementation	16c	Who will generate the allocation sequence, who will enrol participants, and who will assign participants to interventions	______7______
Blinding (masking)	17a	Who will be blinded after assignment to interventions (eg, trial participants, care providers, outcome assessors, data analysts), and how	____7, 8______
	17b	If blinded, circumstances under which unblinding is permissible, and procedure for revealing a participant's allocated intervention during the trial	____7, 9_____
**Methods: Data collection, management, and analysis**			
Data collection methods	18a	Plans for assessment and collection of outcome, baseline, and other trial data, including any related processes to promote data quality (eg, duplicate measurements, training of assessors) and a description of study instruments (eg, questionnaires, laboratory tests) along with their reliability and validity, if known. Reference to where data collection forms can be found, if not in the protocol	_____9______
	18b	Plans to promote participant retention and complete follow-up, including list of any outcome data to be collected for participants who discontinue or deviate from intervention protocols	_____N/A______

For peer review only - http://bmjopen.bmj.com/site/about/guidelines.xhtml

Data management	19	Plans for data entry, coding, security, and storage, including any related processes to promote data quality (eg, double data entry; range checks for data values). Reference to where details of data management procedures can be found, if not in the protocol	_____10_______
Statistical methods	20a	Statistical methods for analysing primary and secondary outcomes. Reference to where other details of the statistical analysis plan can be found, if not in the protocol	___10, 11______
	20b	Methods for any additional analyses (eg, subgroup and adjusted analyses)	____10, 11_____
	20c	Definition of analysis population relating to protocol non-adherence (eg, as randomised analysis), and any statistical methods to handle missing data (eg, multiple imputation)	____10, 11_____
**Methods: Monitoring**			
Data monitoring	21a	Composition of data monitoring committee (DMC); summary of its role and reporting structure; statement of whether it is independent from the sponsor and competing interests; and reference to where further details about its charter can be found, if not in the protocol. Alternatively, an explanation of why a DMC is not needed	____11, 13_____
	21b	Description of any interim analyses and stopping guidelines, including who will have access to these interim results and make the final decision to terminate the trial	____N/A_______
Harms	22	Plans for collecting, assessing, reporting, and managing solicited and spontaneously reported adverse events and other unintended effects of trial interventions or trial conduct	____9_____
Auditing	23	Frequency and procedures for auditing trial conduct, if any, and whether the process will be independent from investigators and the sponsor	____N/A_______
**Ethics and dissemination**			
Research ethics approval	24	Plans for seeking research ethics committee/institutional review board (REC/IRB) approval	_____11_______
Protocol amendments	25	Plans for communicating important protocol modifications (eg, changes to eligibility criteria, outcomes, analyses) to relevant parties (eg, investigators, REC/IRBs, trial participants, trial registries, journals, regulators)	Not included

For peer review only - http://bmjopen.bmj.com/site/about/guidelines.xhtml

Consent or assent	26a	Who will obtain informed consent or assent from potential trial participants or authorised surrogates, and how (see Item 32)	_______6, 7____
	26b	Additional consent provisions for collection and use of participant data and biological specimens in ancillary studies, if applicable	_____N/A_____
Confidentiality	27	How personal information about potential and enrolled participants will be collected, shared, and maintained in order to protect confidentiality before, during, and after the trial	_____9, 10_____
Declaration of interests	28	Financial and other competing interests for principal investigators for the overall trial and each study site	_____13______
Access to data	29	Statement of who will have access to the final trial dataset, and disclosure of contractual agreements that limit such access for investigators	_____11______
Ancillary and post-trial care	30	Provisions, if any, for ancillary and post-trial care, and for compensation to those who suffer harm from trial participation	Not included
Dissemination policy	31a	Plans for investigators and sponsor to communicate trial results to participants, healthcare professionals, the public, and other relevant groups (eg, via publication, reporting in results databases, or other data sharing arrangements), including any publication restrictions	_______11, 12__
	31b	Authorship eligibility guidelines and any intended use of professional writers	_____N/A_____
	31c	Plans, if any, for granting public access to the full protocol, participant-level dataset, and statistical code	____N/A______
**Appendices**			
Informed consent materials	32	Model consent form and other related documentation given to participants and authorised surrogates	Not included
Biological specimens	33	Plans for collection, laboratory evaluation, and storage of biological specimens for genetic or molecular analysis in the current trial and for future use in ancillary studies, if applicable	_____N/A_____

*It is strongly recommended that this checklist be read in conjunction with the SPIRIT 2013 Explanation & Elaboration for important clarification on the items. Amendments to the protocol should be tracked and dated. The SPIRIT checklist is copyrighted by the SPIRIT Group under the Creative Commons "Attribution-NonCommercial-NoDerivs 3.0 Unported" license.

For peer review only - http://bmjopen.bmj.com/site/about/guidelines.xhtml

BMJ Open

The Novel Use of Tranexamic Acid to Reduce the Need for Nasal Packing in Epistaxis (NoPac) Randomised Controlled Trial: research protocol

Journal:	*BMJ Open*
Manuscript ID	bmjopen-2018-026882.R1
Article Type:	Protocol
Date Submitted by the Author:	13-Nov-2018
Complete List of Authors:	Reuben, Adam; Royal Devon & Exeter Hospitals NHS Foundation Trust, Emergency Medicine<br>Appelboam, Andrew; Royal Devon & Exeter Hospital, Emergency Department<br>Barton, Andy; South West Research Design Service,<br>Vickery, Patricia; Plymouth University, Peninsula Clinical Trials Unit<br>Body, Richard; Manchester Royal Infirmary, Emergency Medicine<br>Hilton, Malcolm; Royal Devon and Exeter NHS Foundation Trust, Otolaryngology<br>Coppell, Jason; Royal Devon & Exeter Hospitals NHS Foundation Trust, Haematology<br>Ewings, Paul; Research Design Service, Research Office
**Primary Subject Heading**:	Emergency medicine
Secondary Subject Heading:	Ear, nose and throat/otolaryngology
Keywords:	Epistaxis, Tranexamic acid, Topical, Intranasal, Packing, Emergency

SCHOLARONE™
Manuscripts

For peer review only - http://bmjopen.bmj.com/site/about/guidelines.xhtml

The Novel Use of Tranexamic Acid to Reduce the Need for Nasal Packing in Epistaxis (NoPac) Randomised Controlled Trial: research protocol

Adam Reuben[1], Andrew Appelboam[1], Andy Barton[2], Jane Vickery[3], Richard Body[4], Malcolm Hilton[5], Jason Coppell[6], Paul Ewings[2]

[1]Academic Department of Emergency Medicine, Royal Devon and Exeter Hospital NHS Foundation Trust, Exeter; [2]Research Design Service South West; [3]Peninsula Clinical Trials Unit, Plymouth University;[4]Department of Emergency Medicine, Manchester Royal Infirmary, Manchester; [5]Department of Otolaryngology, Royal Devon and Exeter Hospital, NHS Foundation Trust; [6]Department of Haematology, Royal Devon and Exeter Hospital, NHS Foundation Trust.

Corresponding Author: Adam Reuben.

c/o Emergency Department, Royal Devon & Exeter Hospital NHS Foundation Trust, Barrack Road, Exeter, Devon EX2 5DW

Tel : 00 44 (0)1392 402085

Fax : 00 44 (0)1392 402313

Email: adam.reuben@nhs.net

For peer review only

For peer review only - http://bmjopen.bmj.com/site/about/guidelines.xhtml

Abstract

Introduction: Patients presenting to Emergency Departments (EDs) with epistaxis uncontrolled by subsequent simple first aid measures or application of topical vasoconstrictors will typically undergo anterior nasal packing. Packing is effective, but can be extremely painful and unpleasant and patients usually need hospital admission. Tranexamic Acid (TXA) is a cheap, safe, readily available antifibrinolytic agent known to be beneficial in a variety of clinical settings where uncontrolled bleeding may be a problem. Anecdotal evidence suggests that topical TXA may be of value in persistent epistaxis; however, further evaluation is required.

Methods and analysis: This is a multicentre, double-blind, parallel group, randomised, controlled trial comparing the use of topical intranasal TXA with indistinguishable placebo in adults presenting to UK EDs with persistent atraumatic epistaxis. Follow-up is at one week by structured telephone review. The primary outcome measure is the subsequent need for anterior nasal packing in the ED. Key secondary outcomes include need for hospital admission, blood transfusion and/or further treatment for epistaxis during the index ED attendance. Recruiting 450 patients will provide 90% power to demonstrate an absolute reduction in packing rate from 95% to 85%. An improvement of this magnitude would be of significant benefit to patients and health care providers and justify a change to standard practice. Given the low cost of TXA and its short administration time, a full economic evaluation is not being undertaken.

Ethics and dissemination: The study has been approved by the South West - Bristol Research Ethics Committee (reference 17/SW/0010). We aim to publish the findings in a high impact, international peer reviewed journal. Results will also be shared with the Hereditary Haemorrhagic Telangiectasia foundation (CureHHT) and telangiectasia UK for dissemination through appropriate related forums.

Registration: The study is registered with Current Controlled Trials (ISRCTN34153772) and the EU Clinical Trials Register (EudraCT No: 2016-001530-10).

Key words

Epistaxis; tranexamic acid; topical; intranasal; packing; emergency

For peer review only

For peer review only - http://bmjopen.bmj.com/site/about/guidelines.xhtml

Strengths and limitations of this study

- Atraumatic epistaxis is a common presentation to the Emergency Department
- This is a randomised, double-blind, placebo-controlled study
- The pragmatic study design ensures ease of recruitment and minimal deviation from standard practice
- Recruitment to Emergency Department research studies is notoriously difficult due to workload and staffing pressures
- The wide variety of approaches to treatment in different centres and by different practitioners may lead to difficulties in standardisation of management

Introduction

Epistaxis (nosebleed) is an extremely common condition which causes 60% of the population to seek medical attention at some point in their lives[1]. Patients are frequently elderly, with mean age of over 70 years[2]. In most cases epistaxis resolves with simple measures. These may include firm pressure with the thumb and index finger to the soft anterior part of the nose, the use of ice packs on the bridge of the nose and adopting a forward leaning posture. Many cases, however, are more serious, leading to hospital admission or even, occasionally, death[3]. Current approaches to managing this condition in the Emergency Department (ED) beyond simple first aid, include the use of topical vasoconstrictors, which have been shown to arrest bleeding in up to 65% of patients[4]. Chemical cautery with silver nitrate may also be used but with profuse bleeds it may be difficult to identify the bleeding site and successfully apply cautery[5]. These initial measures may be partially successful and are occasionally repeated to stop the bleeding. If bleeding cannot be stopped with these measures, patients will usually undergo anterior nasal packing.

Whilst effective, nasal packing is recognised to be extremely uncomfortable for patients. The mean pain score associated with insertion of the most commonly used nasal packs (the Merocel) has been shown to be higher than the average pain scores for patients with an acute myocardial infarction (heart attack)[6,7]. Nasal packs typically remain in situ for at least 24 hours, which causes on-going pain (reported mean pain scores 0.5 – 3.5/10) and an uncomfortable sensation of nasal obstruction. One typical published patient story reports, “The packing had my face aching all night”[8]. Removal is also painful with reported pain scores ranging from 0.4 to 7.4. In addition to the pain caused by this procedure, our unpublished feasibility data demonstrate that 86% of patients who undergo anterior nasal packing are admitted to hospital and patients have a mean length of stay of two days. Nasal packing is also associated with a complication rate of up to 28%[9]. Reported complications include infection, toxic shock syndrome, hypoxia, sleep apnoea, pack displacement with airway obstruction, and bleeding on removal.

A recent unpublished national survey (Florey E, Reuben A, Appelboam A, 2014) has shown significant variation in the ED treatments used, which vary according to local protocols, clinician experience, available expertise and departmental workload. Feasibility data at three centres suggest that one third of patients presenting to the ED with epistaxis subsequently require nasal packing.

For peer review only

For peer review only - http://bmjopen.bmj.com/site/about/guidelines.xhtml

TXA is an antifibrinolytic agent which acts by competitively binding to both plasminogen and plasmin in circulating blood at the site of injury, reducing the production and action of plasmin. Plasmin promotes the breakdown of fibrin, a protein that forms the stabilising framework of blood clots, therefore by inhibiting the action of plasmin, clots become more stable. TXA may be used via the intravenous or oral routes, having systemic action, or topically, where its effects are very localised, acting on the mucosa / blood vessels with which it is in contact.

TXA has been used in a variety of clinical and research settings with good evidence of its general efficacy and safety[10]. A large RCT including 20,000 patients showed that intravenous TXA reduces mortality in patients with major trauma and suspected bleeding[11]. Importantly, in this large trial TXA did not cause an increase in thromboembolic complications. In a meta-analysis of 129 trials including 10,488 patients, intravenous TXA was found to reduce mortality and the need for blood transfusion in patients undergoing surgery[12]. No adverse events were noted in any of the patients receiving topical or intravenous treatment. When applied *topically* in patients undergoing surgery, TXA has been shown to reduce bleeding and the need for blood transfusion[10]. The bioavailability of topical TXA is unknown (about 50% via GI tract), but the systemic absorption of a topical dose of 200mg (compared with standard IV dose of 1g) is extremely unlikely to result in significant side effects. Plasma concentrations following topical application are less than one tenth of the level after intravenous administration[10]. Results of an unpublished local study (Florey E, Appelboam A, Reuben A, 2015) to estimate the quantity of TXA absorbed through the nasal mucosa, suggested that of 200mg applied to a dry dental roll, a maximum of 39.8mg is absorbed systemically, with an average of just 14mg. There are no absolute contra-indications to the use of TXA and the large multi-centre RCTs CRASH[11] and HALT-IT[13] have set a precedent with no notable exclusion criteria.

Topical application of tranexamic acid has the potential to inhibit local fibrinolysis at the site of bleeding with minimal systemic absorption[10]. Our systematic review identified that two randomised controlled trials have evaluated topical TXA as a treatment for epistaxis[14]. The first, conducted in 1995, randomised 68 patients to receive topical TXA gel or placebo gel and demonstrated non-significant trends towards lower rates of continued bleeding after 30 minutes and re-bleeding within 30 days[15]. However, the trial was underpowered to detect clinically important differences and significantly more patients who received TXA had presented with moderate or severe bleeds. The second trial randomised 216 patients to receive topical TXA (dental rolls soaked with 500mg in 5ml of TXA) or anterior nasal packing. Significantly fewer patients in the TXA group had active bleeding after 10 minutes with an absolute risk reduction of 40% (number needed to treat 2.5) suggesting substantial benefit. However, this trial was limited by a potential lack of allocation concealment and was unblinded[16]. While they have limitations, these trials suggest that topical TXA is a promising treatment for epistaxis, which could reduce the need for patients to undergo a painful, unpleasant procedure (anterior nasal packing) with its attendant complications and hospital admissions. Topical TXA for persistent epistaxis may therefore be both beneficial for the patient (reduced nasal packing and reduced hospital admission) and for the healthcare system (saving on bed occupancy and providing a cheaper alternative to packing and admission).

For peer review only - http://bmjopen.bmj.com/site/about/guidelines.xhtml

Aims and objectives

The aim of the study is to test the effectiveness of topical intranasal TXA in reducing the need for anterior nasal packing in adult patients presenting to the ED with spontaneous atraumatic epistaxis.

Key objectives are to compare the effect of topical TXA versus placebo on the need for anterior nasal packing, any further treatment in the ED, hospital admission and subsequent length of hospital stay, the requirement for blood products, the re-bleeding rate for patients subsequently discharged from the ED and any adverse events, including thrombotic complications.

Methods and analysis

Study design

This study is a pragmatic 1:1 individually randomised, double-blind, parallel group placebo controlled multi-centre trial. Four hundred and fifty patients presenting to the ED with epistaxis and bleeding that persists after simple first aid measures, followed by standardised topical vasoconstrictor therapy will be randomised to receive either TXA or matched placebo (water for injection), soaked onto a cotton wool dental roll. Outcomes will include anterior nasal packing (primary), any further treatment for epistaxis during the index ED attendance, hospital admission, length of stay, need for blood products and re-bleeding rates for patients discharged home. Thrombotic complications will also be monitored as this is a recognised side effect of TXA, although this is considered a negligible risk with the low dose topical TXA used in this study. Figure 1 illustrates the participant pathway through the trial.

A Standard Operating Procedure (SOP) for the initial management of epistaxis has been implemented at participating sites prior to the study opening to recruitment (Appendix 1). The purpose of this was to standardise i) the application of digital pressure to compress the nose and ii) the use of a topical vasoconstrictor soaked on a cotton wool roll and gently inserted into the bleeding nostril. Whilst not universally used, vasoconstrictor use has been shown to have utility in epistaxis and is advocated in national guidance[6,17]. Participating sites are required to manage potential trial patients according to this standardised procedure for epistaxis management prior to confirming whether patients are eligible for inclusion in the trial. The current protocol is version 1.1, last updated on 2 September 2018.

Study population and setting

The screening and recruitment of patients, delivery of the intervention and recording of outcomes will be carried out within participating UK NHS EDs (Appendix 2).

Screening, recruitment and consent

Adult patients (aged 18 or over) with acute spontaneous epistaxis and continued, uncontrolled bleeding after at least 10 minutes of simple first aid measures (e.g. digital pressure) will be screened for eligibility. Unstable patients or patients with an indication to proceed to immediate nasal packing, will be excluded. Full inclusion and exclusion criteria for the trial are summarised in Table 1.

For peer review only - http://bmjopen.bmj.com/site/about/guidelines.xhtml

Table 1: Inclusion and exclusion criteria

**Inclusion criteria**
Potential participants must satisfy the following criteria to be enrolled in the study:<br>• Aged 18 or over<br>• Presenting to the ED with spontaneous, atraumatic epistaxis, unresolved with simple first aid and standard initial therapy
**Exclusion criteria**
Potential participants meeting any of the following criteria will be excluded from study participation:<br>• Clinical evidence of shock, as determined by the treating clinician, or requirement for resuscitation (including but not limited to systolic BP<90 mmHg).<br>• Known allergy to TXA<br>• Lacking capacity to give consent<br>• Unwilling to give consent<br>• No telephone or unwilling to be contacted by telephone<br>• Known nasopharyngeal, nasal cavity or paranasal malignancy<br>• Pregnancy<br>• Sent to ED for specialist ENT treatment<br>• Already undergone pre-hospital nasal packing<br>• Prior participation in the study (i.e. received allocated treatment)<br>• Prisoners<br>• Epistaxis caused by trauma (excluding simple nose picking)<br>• Known haemophilia

Patients will be screened for eligibility by Good Clinical Practice (GCP) trained staff. ED attendance logs will be screened for the duration of the trial to enable the identification of any missed patients and reporting of simple demographic data to ensure there is no evidence of recruitment bias.

Potentially eligible patients will give written informed consent (supplementary file) during 10 minutes of treatment with a topical vasoconstrictor (e.g. phenylephedrine or dilute adrenaline) soaked on a dental roll and standard nasal compression provided by a proprietary nasal clip, as directed by the SOP provided to participating sites. The written informed consent process will normally be undertaken by the attending clinician but may be delegated to another appropriate GCP-trained member of the research team depending on individual circumstances. Study team members nominated by the Principal Investigator to undertake the consent process will be listed as such on the study delegation log.

Potentially eligible patients will be provided with a concise Participant Information Sheet (PIS), a verbal explanation of the purpose and nature of the trial and a description of what

For peer review only - http://bmjopen.bmj.com/site/about/guidelines.xhtml

participation in the trial will entail. Patients will be given the opportunity to ask questions about the study. Patients providing consent and who continue to bleed after application of topical vasoconstrictor are confirmed eligible and will proceed to randomisation. Informed consent will be sought prior to confirmation of eligibility in order to prevent delay to further treatment should the patient continue to bleed from their nose once the vasoconstrictor-soaked dental roll is removed.

Most bleeding can be at least temporarily controlled with nasal compression sufficient to allow written informed consent. However, witness consent will be permitted for patients who are unable to provide written consent e.g. due to continued active bleeding during nasal compression but who have full capacity and have expressed clear interest in taking part. If a patient agrees to participate, he/she will be asked to complete an Informed Consent Form which will be countersigned by the staff member taking consent. A record of the patient's consent to participate will be documented in the hospital notes where a copy of the completed consent form and participant information sheet will also be filed.

Randomisation and blinding

Treatment allocations will be determined by randomisation stratified by centre using variable sized blocks, from a computer-generated allocation sequence provided by the CTU. Sequentially numbered tamper-evident trial packs will be supplied by the manufacturer containing a sealed vial of the trial solution, two dental rolls and study-specific labels for the trial documentation and patient's notes. Patients will be allocated to receive TXA or placebo by selection of the next sequentially numbered pack. Packs will be signed out by trained staff on the randomisation log. Appropriate sequential use of packs will be strictly audited by the site research teams, trial manager and Trial Management Group.

Consideration was given to the possible need for stratification of patients according to anticoagulant use prior to randomisation. Local audit data from the RD&E hospital have shown that packing rates for those on warfarin or one of the direct oral anti-coagulants (DOACs), as compared with those who are not, are within 5% of each other. Given the extra complexity that stratification would introduce in a time-pressured environment, and the likelihood that randomisation will achieve good balance, no such stratification has been included.

This is a double-blind study hence neither the research teams responsible for treating and assessing patients within this trial, nor the participants themselves, are aware of individual treatment allocations. Should the clinical need arise, a procedure is in place to enable emergency unblinding of treatment allocation.

Trial interventions

Treatments in both arms of the trial are provided by Stockport Pharmaceuticals who are licensed to manufacture products for clinical trials under a Medicines and Healthcare products Regulatory Agency (MHRA) Manufacturers Authorisation (IMP) licence. Participants will receive either:

a) tranexamic acid: presented in a vial containing 4mls of a 100mg/ml solution, enough to saturate two standard cotton wool dental rolls. The standard preparation of TXA solution

For peer review only

For peer review only - http://bmjopen.bmj.com/site/about/guidelines.xhtml

available in the UK contains 500mg of drug in 5mls of clear, colourless solution. A cotton wool roll, such as is routinely used in the management of patients with epistaxis, will absorb approximately 2mls (equivalent to 200mg) of TXA.

b) placebo: an identically presented vial containing 4mls of the excipient used for the active investigational medicinal product (IMP) i.e. water for injection.

Packaging for trial treatments will be identical. A flag label on the vial will be affixed to the trial-specific data collection form for drug accountability purposes. Trial drugs will be stored, at ambient temperature, in a secure drug cupboard within the ED accessible only to trained staff. Approximately 2mls of solution will be used to saturate a cotton wool dental roll which, once saturated, will be inserted into the patient's nose. Sufficient solution will remain in the bottle to allow a second treatment according to the protocol.

The active and placebo solutions will be indistinguishable in appearance, taste and smell to patients and clinicians. All dental rolls will be left in situ for approximately 10 minutes, timed by the research nurse or attending clinician and accompanied by continued standardised nasal compression using a purpose designed disposable nasal clip. This is deemed to be a sufficient period for the formation and stabilisation of blood clots and hence control of bleeding. Patients who deteriorate clinically during this period with uncontrolled bleeding or a fall in blood pressure, or where urgent nasal packing is clinically indicated, will have their dental roll removed and receive further treatment as directed clinically. In our experience this would be a very rare event if the patient was initially stable and eligible for the trial. Such patients will be included in the intention-to-treat analyses.

Participants with on-going bleeding after 10 minutes' treatment with the IMP will undergo an identical second 10 minute treatment using the remaining allocated solution as long as they remain eligible and willing to continue in the study.

Participants who are eligible and randomised but not treated with at least one dose of IMP e.g. because the bleeding stopped between randomisation and administration of IMP, will also be included in the intention-to-treat analyses. However, participants randomised in error and in violation of the protocol e.g. when bleeding was not on-going after vasoconstrictor therapy, will be excluded.

Primary outcome measure

The primary outcome will be the use of anterior nasal packing (of any type) at any time during that ED attendance. The need for packing will be determined after removal of the second trial dental roll by the treating clinician. On-going bleeding will be defined as the presence of visible blood below the nostril. Minor on-going bleeding does not mandate nasal packing and the epistaxis management SOP will advise consideration of further measures such as silver nitrate cautery as appropriate. The decision to pack cannot be strictly defined and must be at the discretion of the clinician based on clinical assessment, but the blinding of clinicians will be sufficiently robust to prevent bias in this decision.

Secondary outcome measures

Secondary outcome measures include hospital admission, need for blood transfusion, recurrent epistaxis and any thrombotic events requiring any hospital re-attendance within

For peer review only - http://bmjopen.bmj.com/site/about/guidelines.xhtml

one week. Any treatment during the index ED episode for continued bleeding and further ED hospital treatments required for epistaxis during the 7 day follow-up period will also be recorded, including details of the type of hospital episode.

Data collection

Data required for the purposes of the trial will be recorded on a study specific case report form (CRF) by the attending clinician, research nurse or other nominated research team member. To save time during the ED attendance, data will be retrospectively recorded in the CRF where applicable. At one week post-attendance, the participant will be contacted by a member of the local research team to collect follow-up information including details of treatment for any further epistaxis and details of any other adverse events experienced since discharge.

Post-intervention management

Following a maximum of two doses of trial treatment, patients will be managed according to local departmental protocols. Any additional treatment(s) will be recorded in the CRF. Prior to discharge, all participants will be given a more detailed information sheet about the study.

Safety monitoring

Extensive evidence supporting the use of both IV TXA (CRASH 2)[11] and topical TXA[10] (Cochrane review) in over 30,000 patients supports an excellent safety profile, with no reported complications in patients receiving topical TXA. The bioavailability of topical TXA is unknown (about 50% via GI tract), but the systemic absorption of a topical dose of 200mg (compared with standard IV dose of 1g) is extremely unlikely to result in significant side-effects. The trial design excludes patients with haemodynamic instability, where the administration of trial treatments could delay urgent life-saving interventions.

Structured telephone review will allow monitoring of all potential complications. Particular attention will be paid to the possibility of thrombotic complications as this is a recognised side effect of both oral and intravenous TXA, although this risk is considered negligible with the low dose topical TXA and the route used in this study.

All serious adverse events (SAEs) will be reported immediately, within 24 hours of discovery, to the CTU who will notify the Chief Investigator. All SAEs will be followed up until resolution. Any SAE considered to be related to trial treatment and not consistent with the information set out in the reference safety information for tranexamic acid will be classified as a suspected unexpected serious adverse reaction (SUSAR) and details passed to the sponsor for immediate unblinding and expedited reporting to the MHRA, as required. In addition to SAEs, non-serious adverse events considered to be possibly, probably or definitely related to trial treatment will be recorded.

A summary report of all serious and non-serious adverse events will be scrutinised by members of the independent Data Monitoring Committee (DMC), Trial Steering Committee and Trial Management Group on a regular basis.

If an individual suffers negligent harm as a result of participating in the trial, NHS indemnity covers NHS staff and those people responsible for conducting the trial who have honorary

For peer review only - http://bmjopen.bmj.com/site/about/guidelines.xhtml

contracts with the relevant NHS Trust. In the case of non-negligent harm, an ex-gratia payment may be considered in the event of a claim. There are no specific provisions for ancillary or post-trial care.

Data handling

Data will be collected and managed in accordance with the Data Protection Act 1998 / General Data Protection Regulation 2018. Each participant will be allocated a unique study number and will then be identified in all study-related documentation by their identification number and initials.

Original CRFs will be posted to the CTU, with copies of the CRF retained at the study site. CRFs will be checked on receipt at CTU and any obvious errors or omissions rectified as far as possible by means of a formalised data query/clarification procedure. All data will be double-entered by CTU staff on to a password-protected SQL Server database and encrypted using Secure Sockets Layer. Double-entered data will be compared for discrepancies using a stored procedure and discrepant data will be verified using the original CRF. After all data cleaning duties have been performed and the database locked, anonymised data will be exported to the trial statistician for blinded analysis.

Determination of sample size

The null hypothesis for the trial is that there is no difference in anterior nasal packing rates for patients with persistent epistaxis after first aid and use of a topical vasoconstrictor, treated with either topical TXA or matched placebo. The patients recruited to the study will have failed simple first aid measures and vasoconstrictor and would ordinarily proceed to packing, according to local protocol. It is likely therefore that the need for packing in the control arm of the trial will be very high; a small number may have their bleeding stopped by the additional 10 minutes of dental roll insertion (albeit soaked in placebo), but we expect about 95% to need packing. We also expect that TXA might produce a large reduction in this need, but given it is inexpensive and safe, even a modest reduction would be worthwhile. A reduction from 95% to 85%, assuming a corrected chi-squared test powered at 90% and with a significance level of 5%, requires 207 patients per group. Because of the nature and timing of the primary outcome, we do not envisage much loss to follow-up or missing data. To be conservative, we aim to recruit a total of 450 patients.

Statistical analysis

A Statistical Analysis Plan will be prepared by the trial statistician and approved by the DMC in advance of any analysis. The trial statistician will conduct the statistical analysis by nominal group (i.e. blinded to treatment allocation) and present the results for interpretation by blinded members of the trial team and oversight committee, prior to disclosure of the treatment allocation and further interpretation of the study findings. There are no pre-planned interim analyses.

CONSORT guidelines[18] will be used to present descriptive data on patients screened, recruited, randomised and treated, and any loss to follow-up. Descriptive data will be presented on baseline characteristics, e.g. demographics. All primary comparative analyses will be based on the intention-to-treat principle, with consideration given to additional sensitivity analyses as appropriate considering per protocol populations (if different) and

For peer review only - http://bmjopen.bmj.com/site/about/guidelines.xhtml

imputation for missing data. The outcomes (including in particular the primary outcome) are binary in nature and will be analysed by mixed effects logistic regression with centre as a random effect. All comparative analyses will be reported with point estimates (odds ratios), 95% confidence intervals and p-values.

Missing data

Given the nature of the short follow-up and limited amount of data to be collected, it is not anticipated that there will be many missing data. Every attempt will be made to retrieve any missing data. Where there data remain missing, sensitivity analyses may be conducted using a range of assumptions where necessary and consideration given to suitable imputation.

Study management and oversight

The study sponsor organisation is the Royal Devon and Exeter NHS Foundation Trust, Barrack Road, Exeter EX2 5DW. Day to day trial management is administered through the UKCRC-registered Peninsula Clinical Trials Unit at Plymouth University. A Trial Management Group including the Chief Investigator, CTU trial managers, trial statistician and other personnel relevant to the study (e.g. clinicians, clinical trials pharmacist, CTU data manager, patient and Sponsor representatives) will meet regularly (usually monthly) throughout the duration of the trial to oversee practical management of the trial.

A Trial Steering Committee (TSC), chaired by an independent member, will oversee the conduct and safety of the trial, ensuring that milestones are achieved and general scientific probity is maintained. An independent Data Monitoring Committee (DMC) will monitor the safety and ethics of the trial by overseeing recruitment, primary outcome data completeness and accumulating safety data.

Patient and public involvement

A Patient Advisory Group was established to contribute to the design of the study from the outset, including input into the proposed trial interventions. This group was made of individuals with prior experience of epistaxis requiring hospital treatment. Further to this, public involvement is maintained through patient representation on the Trial Management Group and TSC. Results will be disseminated to study participants via a website hosted by the CTU and to a wider patient group via CureHHT.

Ethics and dissemination

The trial complies with Declaration of Helsinki and GCP guidelines. The study has been approved by the South West – Central Bristol Research Ethics Committee (REC reference 17/SW/0010) and each of the participating NHS Trust's Research and Development departments. All eligible, willing participants will undergo full written informed consent by GCP-trained staff before taking any part in the study. All protocol modifications will be reviewed by the study sponsor and communicated to relevant parties once approved.

The results will be applicable and of interest to emergency physicians, paramedics, acute physicians, NHS Trusts, general practitioners, emergency care practitioners and patients and efforts will be made to disseminate the information to all of these groups. To that end, the study will be submitted for publication in international, high impact, peer reviewed

For peer review only - http://bmjopen.bmj.com/site/about/guidelines.xhtml

journals whose target audience includes appropriate clinicians. Results will also be shared with the Hereditary Haemorrhagic Telangiectasia (HHT) foundation and telangiectasia UK (patient groups at significant risk of epistaxis) for publication on their websites and presentation through appropriate related forums. After the end of the study, information collected during the study may be made available as an anonymised participant level dataset to other researchers under an appropriate data sharing agreement.

Discussion

The NoPac study is a multi-centre randomised controlled trial designed to investigate the value of topical intranasal tranexamic acid for patients presenting to an ED with epistaxis that persists after simple first aid measures. Such patients frequently progress to requiring anterior nasal packing. Whilst effective, packing is not a benign procedure, can be extremely painful, invariably requires hospital admission and has side-effects and potential significant complications. TXA is safe, cheap and readily available.

A pragmatic design is intended to enhance, rather than impede, the patient journey. The study protocol follows a standard operating procedure for the management of epistaxis, which all recruiting centres have adopted in advance of the trial opening. Given the unpredictable, busy and challanging nature of the ED environment, all appropriate steps have been taken to ensure that the trial processes are simple and straight forward to complete. The trial has been specifically designed to meet its objectives and recruitment targets in this challenging ED setting, using a carefully considered effect size and accrual rate based upon data from participating sites. The trial interventions will not interfere with the standard ED management of epistaxis, so patients will not in anyway be disadvantaged by their inclusion.

If TXA were found to be a useful adjunct to the management of persistent epistaxis, it could obviate the need for nasal packing in those patients where it is effective and reduce the need for hospital admission. This would be an important finding for the benefit of patients and healthcare systems.

Anterior nasal packing is an effective, but uncomfortable method for controlling persistent epistaxis that usually requires hospital admission. Topical TXA has been shown to be safe and effective in a number of settings and there is some evidence that it may be of value in selected patients with epistaxis, although the question is yet to be fully evaluated. The results of this study could realistically lead to a reduction in the need for anterior nasal packing and thus hospital admission for patients with epistaxis and the reduction in for an uncomfortable procedure with recognised complications.

Acknowledgments

We would like to acknowledge and thank Wendy Ingram and Alison Jeffery, Peninsula Clinical Trials Unit, for all their hard work and administrative skills in support of this project and Ian Veitch, our patient representative, for his help and advice since the study start.

Funding

This research is funded by the NIHR Research for Patient Benefit Programme (project number PB-PG-0215-36142). The views expressed are those of the author(s) and not

For peer review only - http://bmjopen.bmj.com/site/about/guidelines.xhtml

necessarily those of the NHS, the NIHR or the Department of Health. The funder has no role in study design or the collection, management, analysis or interpretation of data.

Trial Management Group

Dr Adam Reuben (Chief Investigator); Mr Andy Barton (Associate Professor in Healthcare Research Methodology, Plymouth University); Prof Andrew Appelboam (Co-applicant, Honorary Associate Clinical Professor, University of Exeter Medical School), Professor Paul Ewings (Trial Statistician; Director, SW Research Design Service), Ms Jane Vickery (Senior Trial Manager, Peninsula Clinical Trials Unit), Dr Jason Coppell (Co-applicant, Consultant Haematologist, Royal Devon & Exeter NHS Foundation Trust), Mr Malcolm Hilton (Co-applicant, Consultant Otolaryngologist, Royal Devon & Exeter NHS Foundation Trust), Prof Richard Body (Co-applicant, Professor of Emergency Medicine, Central Manchester University Hospitals NHS Foundation Trust, Dr Alison Kerridge (Sponsor representative, Royal Devon & Exeter NHS Foundation Trust), Mrs Fiona Walters (Pharmacy Clinical Trials Manager, Royal Devon & Exeter NHS Foundation Trust), Mrs Shirley Todd (pharmacist), Mr Ian Veitch (Patient and Public Involvement representative), Gayle Githens-Mazer (Research Delivery Manager, NIHR Clinical Research Network South West Peninsula).

Trial Steering Committee

Chair, Prof Jonathan Benger (Consultant in Emergency Medicine, University Hospitals Bristol NHS Foundation Trust); Dr Adam Reuben (Chief Investigator, Consultant in Emergency Medicine, Royal Devon & Exeter Hospital); Mr Richard Williams (Specialist Registrar in Ear, Nose and Throat Surgery, Bristol Royal Infirmary) Dr Fiona Warren (Research Fellow in Statistics, University of Exeter Medical School), Prof Andrew Green (Patient and Public Involvement Representative)

Independent Data Monitoring Committee

Professor Gordon Taylor; Dr Tim Nokes (Consultant Haematologist, Derriford Hospital, Plymouth); Professor Tim Coates (Consultant Emergency Medicine, Leicester Royal Infirmary; Professor Paul Ewings (Research Design Service SW)

Competing Interests

There are no competing interests to declare.

Contributorship statement

All authors were co-applicants on the NIHR RfPB grant application, helped with the design of the study and have contributed to successive drafts of this paper.

AR is the Chief Investigator, provides clinical expertise and was responsible for conceiving the study and contributing to its initial design.

AA provides clinical expertise, contributed to the initial design of the study and supports the ongoing management of the study.

JV is the trial manager, responsible for overseeing the day-to-day running of the study.

PE is the trial statistician and provided expertise in the overall design of the study.

For peer review only - http://bmjopen.bmj.com/site/about/guidelines.xhtml

RB provides clinical expertise, contributed to the initial design of the study and supports the ongoing management of the study.

MH provides clinical expertise in otolaryngology and contributes to study management.

JC provides clinical expertise in haematology and contributes to study management.

AB provides methodological expertise and contributes to study management.

Representatives of the study sponsor attend trial management meetings and have reviewed the study protocol.

For peer review only

For peer review only - http://bmjopen.bmj.com/site/about/guidelines.xhtml

References

1. Pope LER, Hobbs CGL. Epistaxis: an update on current management. Postgrad Med J 2005;81:309-314

2. Traboulsi H, Alam E, Hadi U. Changing Trends in the Management of Epistaxis. *Int J Otolaryngol*. 2015. 2015:263987

3. Tan LK, Calhoun KH. Epistaxis. *Med Clin North Am*. 1999;83:43–56

4. Krempl, G. A. and A. D. Noorily (1995). Use of oxymetazoline in the management of epistaxis. Ann Otol Rhinol Laryngol 104(9 Pt 1): 704-6

5. Barr GD. Silver nitrate cautery and epistaxis. Arch Emerg Med 1989;6:233

6. Pringle MB, Beasley P, Brightwell AP. The use of Merocel nasal packs in the treatment of epistaxis. J Laryngol Otol 2006:96;543-546

7. Herlitz J, Richterova A, Bondestam E. Chest pain in acute myocardial infarction: a descriptive study according to subjective assessment and morphine requirement. Clin Cardiol 1986;9:423-428

8. http://www.emedicinehealth.com/nosebleeds/viewer-comments_em-74-page3.htm emedicine health viewer comments

9. Germann CA, Southall JC. Management of epistaxis and complications associated with anterior nasal packing. Ann Emerg Med 2004;44:s43-s44

10. Ker K, Beecher D, Roberts I. Topical application of tranexamic acid for the reduction of bleeding (review). Cochrane Database Syst Rev 2013;7: CD010562

11. The CRASH-2 Study. The CRASH-2 Collaborators. Lancet 2010;377:1096-1101

12. Ker K, Prieto-Merino D, Roberts I. Systematic review, meta-analysis and meta-regression of the effect of tranexamic acid on surgical blood loss. B J Surg 2013;100(10)

13. Roberts I, Coates T, Edwards P. HALT-IT - tranexamic acid for the treatment of gastrointestinal bleeding: study protocol for a randomised controlled trial Published online 2014 Nov 19. doi: 10.1186/1745-6215-15-450

14. Hilton L, Reuben AD. Topical intranasal tranexamic acid for spontaneous epistaxis. BESTBETS Best evidence topic reviews http://bestbets.org/bets/bet.php?id=2569

15. Tibbelin A, Aust R, Bende M et al. Effect of local tranexamic acid gel in the treatment of epistaxis ORL 1995;57:207-209

16. Zahed R, Moharamzadeh P, AlizadehArasi S. A new and rapid method for epistaxis treatment using injectable form of tranexamic acid topically: a randomized controlled trial 2013;31:1389-1392

17. https://bestpractice.bmj.com/topics/en-gb/421?q=Epistaxis&c=suggested

18. http://www.consort-statement.org/

For peer review only - http://bmjopen.bmj.com/site/about/guidelines.xhtml

Legend

Table 1 Inclusion and exclusion criteria

Figure 1 Participant pathway

Appendix 1 Epistaxis Standard Operating Procedure

Appendix 2 NoPac study sites

For peer review only

For peer review only - http://bmjopen.bmj.com/site/about/guidelines.xhtml

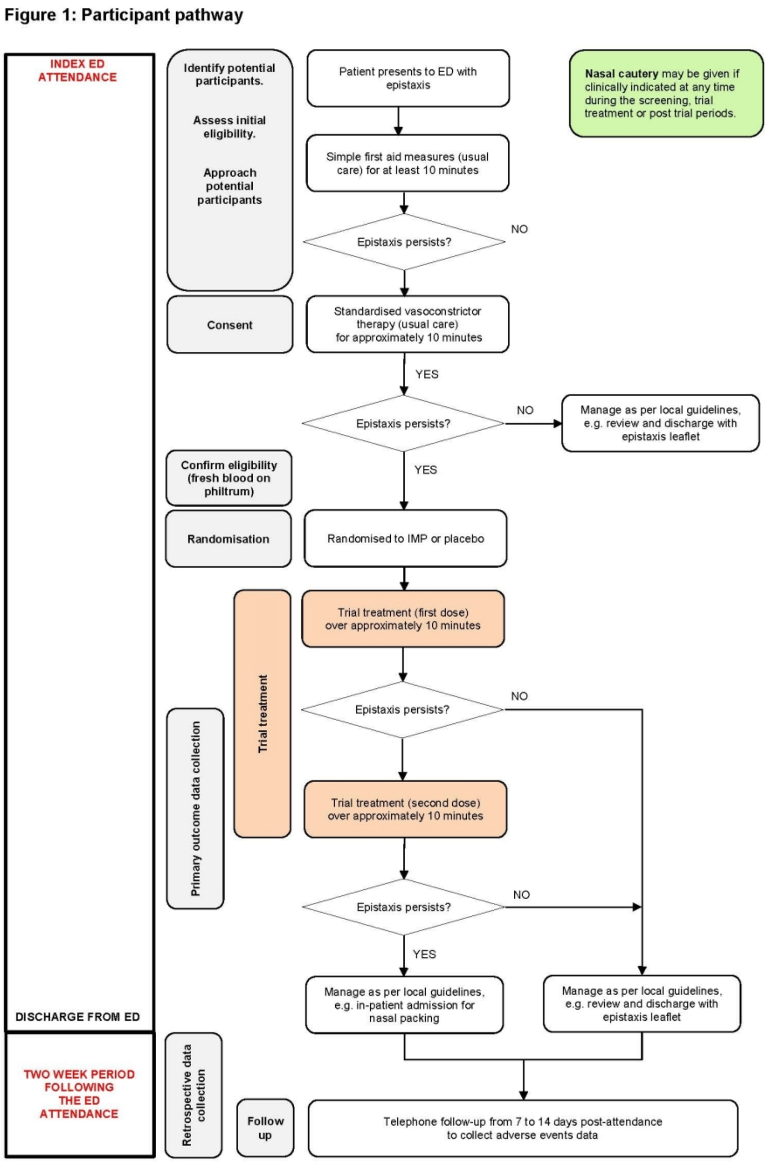

Figure 1: Participant pathway

209x297mm (200 x 200 DPI)

NHS Trust Header

Participant study number: ☐☐☐☐☐

Principal Investigator: Dr <PI name>

PARTICIPANT CONSENT FORM

NoPac study: Novel use of tranexamic acid to reduce the need for nasal packing

Please initial each box

1. I confirm that I have read the information sheet (version 2.0 dated 20.02.2017) for the above study. I have had the opportunity to consider the information, ask questions and have had these answered satisfactorily. ☐

2. I understand that my participation in this study is voluntary and that I am free to withdraw at any time without giving any reason and without my medical care or legal rights being affected. ☐

3. I understand that relevant sections of my medical notes and information collected during the study may be looked at by individuals from the Sponsor organisation or its' representatives, my local NHS Trust and the regulatory authorities, where it is relevant to my taking part in this research. I give permission for these individuals to have access to my records. ☐

4. I agree to my General Practitioner being informed of my participation in the study. ☐

5. I agree that my telephone contact details may be temporarily stored at my local NHS Trust to allow the study team to contact me after one week. ☐

6. I consent to take part in this study. ☐

Print name (Participant)	Date	Signature

IF PARTICIPANT IS UNABLE TO COMPLETE THIS FORM, ASK WITNESS TO COMPLETE THE FOLLOWING:

Print name (Witness)	Date	Signature

Relationship of witness to participant: ____________________

Print name (Person taking consent)	Date	Signature

For peer review only - http://bmjopen.bmj.com/site/about/guidelines.xhtml

When completed: 1 (original) for researcher site file; 1 copy for participant; 1 copy for medical notes

Appendix 1: Epistaxis Standard Operating Procedure (SOP)

This haemostasis SOP is for use in cooperative, haemodynamically stable patients with *ongoing* bleeding. If in doubt, get senior help. See full ED guideline for details.

STEP 1
Ensure appropriate simple first aid measures have been tried. These have usually been undertaken pre-hospital or after triage.

↓

STEP 2
Clear any large blood clot from the nasal passages by gentle nose blowing, suction or with nasal forceps and examine the nose.

↓

STEP 3
If active bleeding, insert a dental roll soaked in **Lignocaine and Phenylephrine*** topical solution in the anterior nose and ask the patient to compress the nose over Little's area (soft part of nose) for 10 minutes with firm digital pressure.

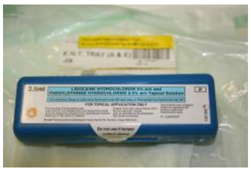

↓ 10 mins

STEP 4
Re-examine nose ideally with a nasal speculum and good light. Cauterize any visible culprit vessel in Little's area with silver nitrate.
Note: Generally significant bleeding needs to be controlled before cautery.
Minor continued bleeding may require further period of compression with the Lignocaine and Phenylephrine topical solution soaked roll.

↓

STEP 5
If on-going brisk bleeding despite above steps anterior pack the nose with either a rapid rhino or nasal tampon and refer to ENT

*or lignocaine and adrenaline or an alternative topical vasoconstrictor (according to preferred local protocols)

For peer review only - http://bmjopen.bmj.com/site/about/guidelines.xhtml

Appendix 2: NoPac study sites

Site Name
Royal Devon & Exeter Hospital, Exeter
Derriford Hospital, Plymouth
Royal United Hospital, Bath
Manchester Royal Infirmary
Gloucestershire Royal Hospital
Cheltenham General Hospital
Southmead Hospital, Bristol
North Devon District Hospital, Barnstaple
Musgrove Park Hospital, Taunton
Salford Royal Hospital
Royal Derby Hospital
Royal Infirmary of Edinburgh
Dorset County Hospital, Dorchester
Royal Cornwall Hospital, Truro
Norfolk & Norwich University Hospital, Norwich
Yeovil District Hospital
St George's Hospital, London
St Thomas' Hospital, London
John Radcliffe Hospital, Oxford
Barts Health NHS Trust: Royal London Hospital
Barts Health NHS Trust: Whipps Cross Hospital
Epsom & St Helier NHS Trust: Epsom Hospital
Epsom & St Helier NHS Trust: St Helier Hospital
Addenbrooke's Hospital, Cambridge
Royal Berkshire Hospital, Reading
University Hospitals Coventry & Warwickshire

For peer review only

SPIRIT

STANDARD PROTOCOL ITEMS: RECOMMENDATIONS FOR INTERVENTIONAL TRIALS

SPIRIT 2013 Checklist: Recommended items to address in a clinical trial protocol and related documents*

Section/item	Item No	Description	Addressed on page number
**Administrative information**			
Title	1	Descriptive title identifying the study design, population, interventions, and, if applicable, trial acronym	______1______
Trial registration	2a	Trial identifier and registry name. If not yet registered, name of intended registry	______2______
	2b	All items from the World Health Organization Trial Registration Data Set	____________
Protocol version	3	Date and version identifier	Not included
Funding	4	Sources and types of financial, material, and other support	______12_____
Roles and responsibilities	5a	Names, affiliations, and roles of protocol contributors	____1, 13-14__
	5b	Name and contact information for the trial sponsor	_____11______
	5c	Role of study sponsor and funders, if any, in study design; collection, management, analysis, and interpretation of data; writing of the report; and the decision to submit the report for publication, including whether they will have ultimate authority over any of these activities	__12, 13, 14___
	5d	Composition, roles, and responsibilities of the coordinating centre, steering committee, endpoint adjudication committee, data management team, and other individuals or groups overseeing the trial, if applicable (see Item 21a for data monitoring committee)	_____11, 13___

For peer review only - http://bmjopen.bmj.com/site/about/guidelines.xhtml

Introduction

Background and rationale	6a	Description of research question and justification for undertaking the trial, including summary of relevant studies (published and unpublished) examining benefits and harms for each intervention	_____3, 4_____
	6b	Explanation for choice of comparators	_____5_______
Objectives	7	Specific objectives or hypotheses	______5______
Trial design	8	Description of trial design including type of trial (eg, parallel group, crossover, factorial, single group), allocation ratio, and framework (eg, superiority, equivalence, noninferiority, exploratory)	______5______

Methods: Participants, interventions, and outcomes

Study setting	9	Description of study settings (eg, community clinic, academic hospital) and list of countries where data will be collected. Reference to where list of study sites can be obtained	_____5_______
Eligibility criteria	10	Inclusion and exclusion criteria for participants. If applicable, eligibility criteria for study centres and individuals who will perform the interventions (eg, surgeons, psychotherapists)	______6______
Interventions	11a	Interventions for each group with sufficient detail to allow replication, including how and when they will be administered	______7,8______
	11b	Criteria for discontinuing or modifying allocated interventions for a given trial participant (eg, drug dose change in response to harms, participant request, or improving/worsening disease)	______8_______
	11c	Strategies to improve adherence to intervention protocols, and any procedures for monitoring adherence (eg, drug tablet return, laboratory tests)	_____N/A______
	11d	Relevant concomitant care and interventions that are permitted or prohibited during the trial	_____N/A______
Outcomes	12	Primary, secondary, and other outcomes, including the specific measurement variable (eg, systolic blood pressure), analysis metric (eg, change from baseline, final value, time to event), method of aggregation (eg, median, proportion), and time point for each outcome. Explanation of the clinical relevance of chosen efficacy and harm outcomes is strongly recommended	______8,9______
Participant timeline	13	Time schedule of enrolment, interventions (including any run-ins and washouts), assessments, and visits for participants. A schematic diagram is highly recommended (see Figure)	______6 -9____

For peer review only - http://bmjopen.bmj.com/site/about/guidelines.xhtml

Sample size	14	Estimated number of participants needed to achieve study objectives and how it was determined, including clinical and statistical assumptions supporting any sample size calculations	_____10______
Recruitment	15	Strategies for achieving adequate participant enrolment to reach target sample size	Not included
**Methods: Assignment of interventions (for controlled trials)**			
Allocation:			
Sequence generation	16a	Method of generating the allocation sequence (eg, computer-generated random numbers), and list of any factors for stratification. To reduce predictability of a random sequence, details of any planned restriction (eg, blocking) should be provided in a separate document that is unavailable to those who enrol participants or assign interventions	_____7_______
Allocation concealment mechanism	16b	Mechanism of implementing the allocation sequence (eg, central telephone; sequentially numbered, opaque, sealed envelopes), describing any steps to conceal the sequence until interventions are assigned	_____7_______
Implementation	16c	Who will generate the allocation sequence, who will enrol participants, and who will assign participants to interventions	______7______
Blinding (masking)	17a	Who will be blinded after assignment to interventions (eg, trial participants, care providers, outcome assessors, data analysts), and how	____7, 8______
	17b	If blinded, circumstances under which unblinding is permissible, and procedure for revealing a participant's allocated intervention during the trial	____7, 9_____
**Methods: Data collection, management, and analysis**			
Data collection methods	18a	Plans for assessment and collection of outcome, baseline, and other trial data, including any related processes to promote data quality (eg, duplicate measurements, training of assessors) and a description of study instruments (eg, questionnaires, laboratory tests) along with their reliability and validity, if known. Reference to where data collection forms can be found, if not in the protocol	_____9______
	18b	Plans to promote participant retention and complete follow-up, including list of any outcome data to be collected for participants who discontinue or deviate from intervention protocols	_____N/A______

For peer review only - http://bmjopen.bmj.com/site/about/guidelines.xhtml

Data management	19	Plans for data entry, coding, security, and storage, including any related processes to promote data quality (eg, double data entry; range checks for data values). Reference to where details of data management procedures can be found, if not in the protocol	_____10_______
Statistical methods	20a	Statistical methods for analysing primary and secondary outcomes. Reference to where other details of the statistical analysis plan can be found, if not in the protocol	___10, 11______
	20b	Methods for any additional analyses (eg, subgroup and adjusted analyses)	____10, 11_____
	20c	Definition of analysis population relating to protocol non-adherence (eg, as randomised analysis), and any statistical methods to handle missing data (eg, multiple imputation)	____10, 11_____
**Methods: Monitoring**			
Data monitoring	21a	Composition of data monitoring committee (DMC); summary of its role and reporting structure; statement of whether it is independent from the sponsor and competing interests; and reference to where further details about its charter can be found, if not in the protocol. Alternatively, an explanation of why a DMC is not needed	____11, 13_____
	21b	Description of any interim analyses and stopping guidelines, including who will have access to these interim results and make the final decision to terminate the trial	____N/A_______
Harms	22	Plans for collecting, assessing, reporting, and managing solicited and spontaneously reported adverse events and other unintended effects of trial interventions or trial conduct	____9_____
Auditing	23	Frequency and procedures for auditing trial conduct, if any, and whether the process will be independent from investigators and the sponsor	____N/A_______
**Ethics and dissemination**			
Research ethics approval	24	Plans for seeking research ethics committee/institutional review board (REC/IRB) approval	_____11_______
Protocol amendments	25	Plans for communicating important protocol modifications (eg, changes to eligibility criteria, outcomes, analyses) to relevant parties (eg, investigators, REC/IRBs, trial participants, trial registries, journals, regulators)	Not included

For peer review only - http://bmjopen.bmj.com/site/about/guidelines.xhtml

Consent or assent	26a	Who will obtain informed consent or assent from potential trial participants or authorised surrogates, and how (see Item 32)	_______6, 7____
	26b	Additional consent provisions for collection and use of participant data and biological specimens in ancillary studies, if applicable	______N/A______
Confidentiality	27	How personal information about potential and enrolled participants will be collected, shared, and maintained in order to protect confidentiality before, during, and after the trial	______9, 10______
Declaration of interests	28	Financial and other competing interests for principal investigators for the overall trial and each study site	______13______
Access to data	29	Statement of who will have access to the final trial dataset, and disclosure of contractual agreements that limit such access for investigators	______11______
Ancillary and post-trial care	30	Provisions, if any, for ancillary and post-trial care, and for compensation to those who suffer harm from trial participation	Not included
Dissemination policy	31a	Plans for investigators and sponsor to communicate trial results to participants, healthcare professionals, the public, and other relevant groups (eg, via publication, reporting in results databases, or other data sharing arrangements), including any publication restrictions	_______11, 12__
	31b	Authorship eligibility guidelines and any intended use of professional writers	______N/A______
	31c	Plans, if any, for granting public access to the full protocol, participant-level dataset, and statistical code	____N/A______
**Appendices**			
Informed consent materials	32	Model consent form and other related documentation given to participants and authorised surrogates	Not included
Biological specimens	33	Plans for collection, laboratory evaluation, and storage of biological specimens for genetic or molecular analysis in the current trial and for future use in ancillary studies, if applicable	______N/A______

*It is strongly recommended that this checklist be read in conjunction with the SPIRIT 2013 Explanation & Elaboration for important clarification on the items. Amendments to the protocol should be tracked and dated. The SPIRIT checklist is copyrighted by the SPIRIT Group under the Creative Commons "Attribution-NonCommercial-NoDerivs 3.0 Unported" license.

For peer review only - http://bmjopen.bmj.com/site/about/guidelines.xhtml
