# Supplementary material for: Novel use of tranexamic acid to reduce the need for Nasal Packing in Epistaxis (NoPac) randomised controlled trial: research protocol
Source: BMJ Open. 2019 Feb 15;9(2):e026882. doi: 10.1136/bmjopen-2018-026882 (PMC6398761; doi:10.1136/bmjopen-2018-026882)
Supplement: Reviewer comments [file bmjopen-2018-026882.reviewer_comments.pdf]

PEER REVIEW HISTORY

BMJ Open publishes all reviews undertaken for accepted manuscripts. Reviewers are asked to complete a checklist review form (**http://bmjopen.bmj.com/site/about/resources/checklist.pdf**) and are provided with free text boxes to elaborate on their assessment. These free text comments are reproduced below.

ARTICLE DETAILS

**TITLE (PROVISIONAL)**	The Novel Use of Tranexamic Acid to Reduce the Need for Nasal Packing in Epistaxis (NoPac) Randomised Controlled Trial: research protocol
**AUTHORS**	Reuben, Adam; Appelboam, Andrew; Barton, Andy; Vickery, Patricia; Body, Richard; Hilton, Malcolm; Coppell, Jason; Ewings, Paul

This article was not externally reviewed at BMJ Open. Protocols that have been independently assessed prior to submission to BMJ Open are usually fast-tracked to publication on the grounds that further substantial changes will not be possible. This independent assessment will usually be external, independent review for both a substantial grant award from a non-commercial or government funder and ethics approval.
